# Double N-arylation reaction of polyhalogenated 4,4’-bipyridines. Expedious synthesis of functionalized 2,7-diazacarbazoles

**DOI:** 10.3762/bjoc.8.26

**Published:** 2012-02-14

**Authors:** Mohamed Abboud, Emmanuel Aubert, Victor Mamane

**Affiliations:** 1 Structure et Réactivité des Systèmes Moléculaires Complexes (SRSMC), UMR CNRS-UHP 7565 Nancy Université, BP 70239, Bd des Aiguillettes, 54506 Vandoeuvre-les-Nancy, France; 2 Cristallographie, Résonance Magnétique et Modélisations (CRM2), UMR CNRS-UHP 7036 Nancy Université, BP 70239, Bd des Aiguillettes, 54506 Vandoeuvre-les-Nancy, France

**Keywords:** 4,4’-bipyridine, cross-coupling, crystal packing, diazacarbazole, X-ray diffraction

## Abstract

Unusual 2,7-diazacarbazoles were prepared in one step from readily available tetra-halogenated 4,4’-bipyridines by using a double N-arylation reaction in the presence of the Pd–XPhos catalyst system. Moderate to good yields were obtained in this site-selective Buchwald–Hartwig double amination. The functionalization of these tricyclic derivatives was performed by using Pd-catalyzed cross-coupling reactions such as the Stille and Suzuki couplings. Two compounds were analyzed by X-ray diffraction and show π–π stacking involving the diazacarbazole moieties and the phenyl rings of functionalized groups.

## Introduction

Only a few examples of the preparation of diazacarbazoles have been reported [1–5] and up to date no general method is available in the literature. However, these diaza analogues of carbazoles [6] have shown interesting biological [7–10] and photophysical [11–13] properties and have been used as ligands in catalysis [14].

The double palladium-catalyzed N-arylation strategy for the synthesis of carbazoles has been extensively used in the literature [15–22]. The methodology was further applied for the preparation of dithienopyrroles [23] and dibenzothienopyrroles [24]. One example was found in a patent concerning the synthesis of 3,6-diazacarbazole by using a palladium-catalyzed double N-arylation of 4,4’-dichloro-3,3’-bipyridine, itself obtained after a long reaction sequence [25–27]. In another patent, an intramolecular Buchwald–Hartwig amination [28–29] was used to generate a 2,7-diazacarbazole derivative in low yield [30].

**Scheme 1:**
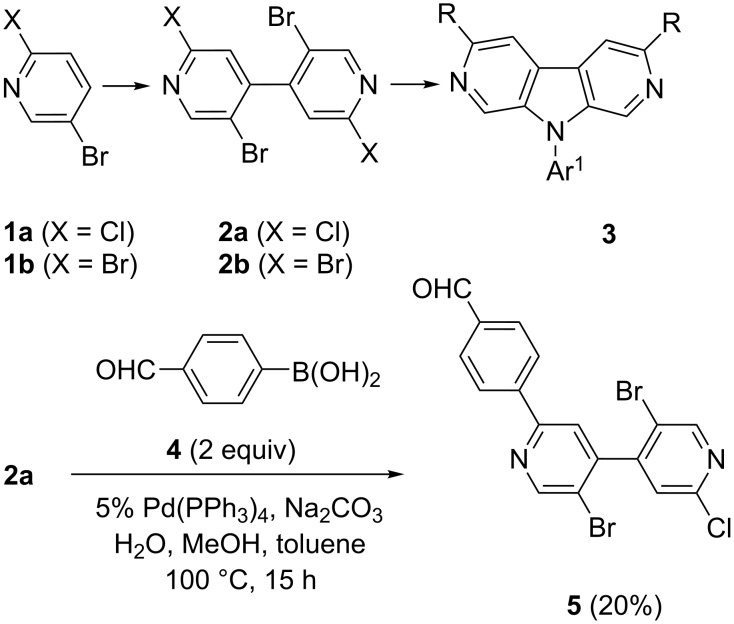
Cross-coupling reactions of bipyridines **2**.

Recently, we reported a one-step preparation of tetrahalogenated 4,4’-bipyridines **2** starting from 2,5-dihalopyridines **1** [31]. Herein, we report on the synthesis of functionalized 2,7-diazacarbazoles **3** through the double N-arylation of **2**. Considering compound **2a**, the C–Br over C–Cl regioselectivity was not obvious since under Suzuki conditions with 2 equiv of 4-formylbenzeneboronic acid (**4**), compound **5**, resulting from a C–Cl functionalization, was obtained as the major product from a complex mixture (Scheme 1) [32].

## Results and Discussion

**Scheme 2:**
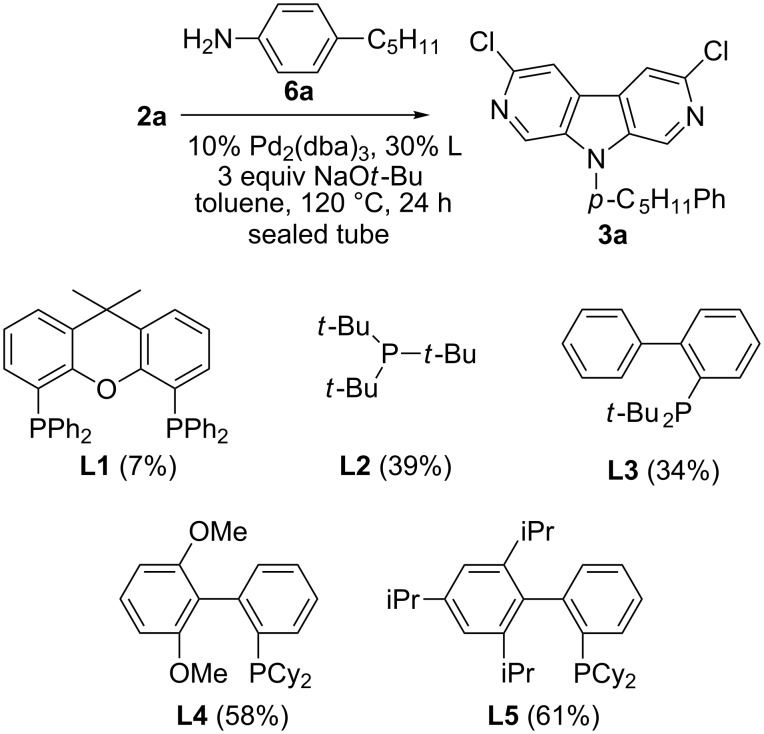
Ligand effect in the double N-arylation of **2a** with **6a**.

The efficiency of the double amination reaction of **2a** with 4-pentylaniline (**6a**) was investigated with several ligands in toluene at 120 °C, and in the presence of Pd_2_(dba)_3_ as the source of metal catalyst (Scheme 2). Ligands such as Xantphos [33], biaryl monophosphines [34] and *t*-Bu_3_P [35] were tested since they were found to be very effective in the selective C-5 amination of 2-chloro-5-bromopyridines [36–37]. The use of the chelating Xantphos ligand (**L1**) led to a poor yield of **3a**, whereas *t*-Bu_3_P (**L2**) and JohnPhos (**L3**) afforded product **3a** with a moderate yield. The more hindered monophosphine ligands SPhos (**L4**) and XPhos (**L5**) showed good results, with an optimum yield of 61% achieved with XPhos [38]. The bulk of the phosphine ligand enhances the stability and the activity of the catalytic system [39] thus allowing the use of an elevated temperature (120 °C) necessary for the double N-arylation reaction [20].

**Figure 1:**
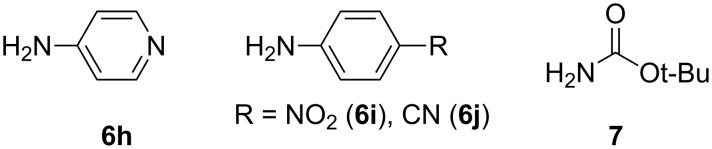
Unsuccessful substrates in the double N-arylation of **2a**.

Encouraged by these results, we further explored the scope and limitations of the Pd_2_(dba)_3_–XPhos catalyst system (Table 1). Under the standard conditions summarized in Table 1 (footnote a), the double N-arylation of **2a** with various aromatic amines **6** furnished 2,7-diazacarbazoles **3** with good chemoselectivities. Moderate to good yields were generally obtained in this reaction (Table 1, entries 1–6). Aniline **6g** bearing an electron withdrawing trifluoromethyl group led to a low yield of **3g** (Table 1, entry 7) whereas no cyclized product was observed with other electron-deficient anilines **6h**–**j** and carbamate **7** (Figure 1). Other methods to react **7** with bipyridine **2a** were tested without success, including the use of Cs_2_CO_3_ in dioxane [40] instead of *t*-BuONa in toluene, and the CuI–DMEDA catalyzed reaction [41].

**Table 1:** Double N-arylation of **2a** with aromatic amines **6** catalyzed by a palladium–XPhos complex.^a^

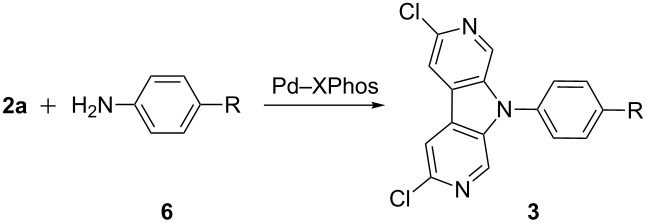				

entry	R	**6**	**3**	yield (%)

1	C_5_H_11_	**6a**	**3a**	61
2	OMe	**6b**	**3b**	55
3	SMe	**6c**	**3c**	50
4	H	**6d**	**3d**	61
5	Cl	**6e**	**3e**	56
6	F	**6f**	**3f**	49
7	CF_3_	**6g**	**3g**	29

^a^General reaction conditions: **2a** (0.26 mmol), **6** (0.234 mmol), Pd_2_(dba)_3_ (0.026 mmol), XPhos (0.078 mmol), *t*-BuONa (0.78 mmol), toluene (0.2 M), 120 °C, sealed tube.

**Scheme 3:**
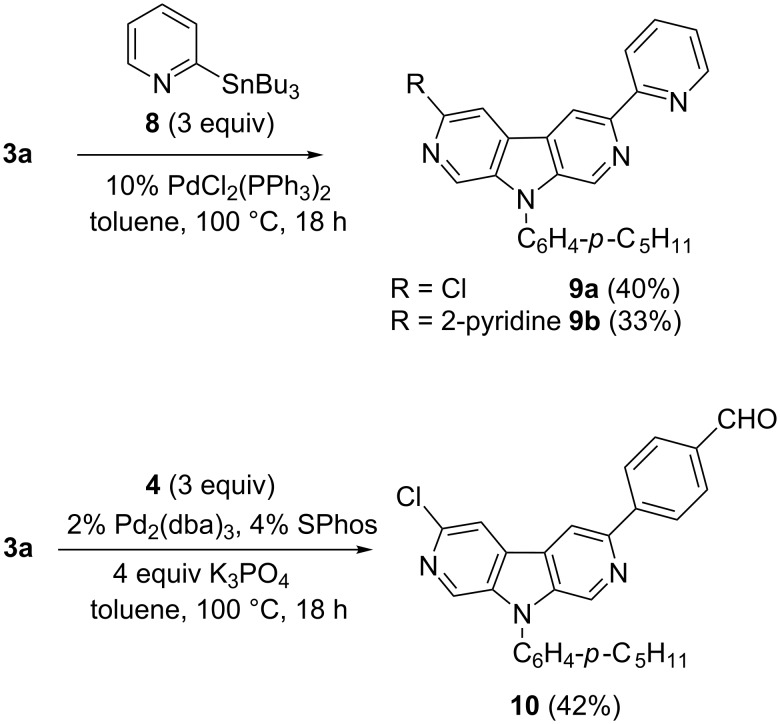
Functionalization of diazacarbazole **2a**.

We then turned our attention to the preparation of functionalized diazacarbazoles **3** by means of cross-coupling reactions. Initial experiments with 2,7-diazacarbazole **3a** showed a rather low reactivity of the C–Cl bonds in the 3- and 6-positions. Indeed, the Stille coupling [42] of **3a** with 2-tributylstannylpyridine (**8**) afforded a mixture of mono- and di-functionalized compounds **9a** and **9b** in 40% and 33% yields, respectively. The Suzuki coupling [43] of **3a** with boronic acid **4** was less efficient giving only the mono-functionalized compound **10** in 42% yield (Scheme 3) while no conversion was observed with 4-methylthiobenzene boronic acid.

**Scheme 4:**
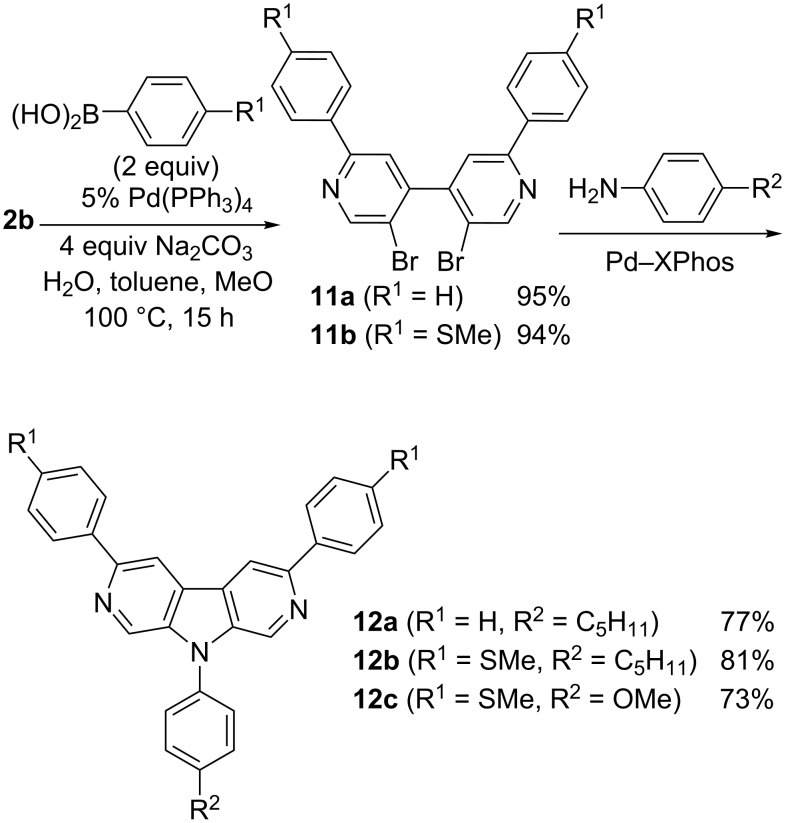
Functionalized diazacarbazoles **12a**–**c** from bipyridine **2b**.

Better results were obtained by means of a reverse sequence. First, the C-2- and C-2’-positions of bipyridine **2b** were functionalized by a selective Suzuki coupling to give the new bipyridine derivatives **11a**,**b**, which underwent the double N-arylation to afford 3,6-difunctionalized 2,7-diazacarbazoles **12a**–**c** in good overall yields (Scheme 4).

Single crystals of compounds **3b** and **12c** were obtained from evaporation of *n*-hexane and chloroform solutions, respectively. Whereas **3b** crystallizes with one molecule per asymmetric unit in space group *C*2/*c*, the more functionalized **12c** diazacarbazole is described by the less symmetric *P*−1 space group with two crystallographically independent molecules (Supporting Information File 1). In both compounds the diazacarbazole moieties are almost, but not rigorously, planar, as evidenced by the dihedral angles between the pyridyl rings (2.86(9)° for **3b**; 2.09(14)° and 3.15(14)° for **12c**). Due to steric hindrance, the methoxyphenyl groups are twisted from the diazacarbazole mean plane (dihedral angles of 50.90(4)° for **3b**, 59.00(6)° and 48.45(5)° for **12c**). On the opposite, the methylsulfanylphenyl groups in **12c** are almost coplanar with the diazacarbazole moiety (dihedral angles of 2.17(15)°/13.92(11)° and 8.30(14)°/13.86(11)°).

**Figure 2:**
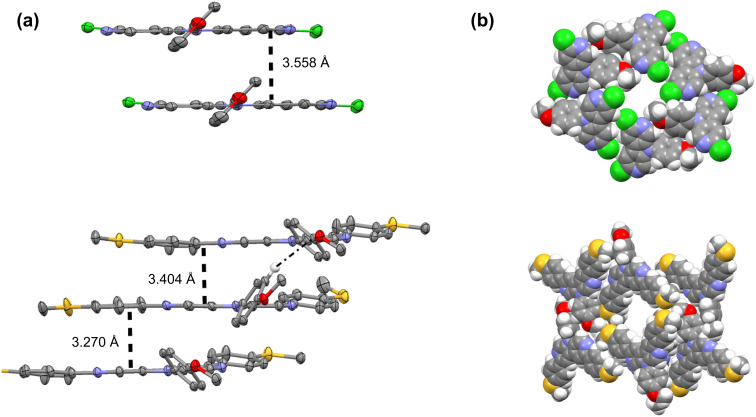
**(**a) ORTEP views showing the π–π (dashed lines) and selected C–H···π (dotted-dashed line) interactions in **3b** (up) and **12c** (down). Atomic displacement ellipsoids are drawn at the 50% level of probability; all other hydrogen atoms are omitted for clarity. Disordered solvent molecules are not shown. (b) Space-filling views showing the channels along [010] in **3b** (up) and [100] in **12c** (down). Disordered solvent molecules occupying these channels are not shown.

In both compounds the crystal packing is governed by π–π stacking, forming infinite columns in which molecules interact through diazacarbazole moieties (interplanar distances are 3.558 Å for **3b**, 3.270 Å and 3.404 Å for **12c**). The methoxyphenyl group is also involved in this π–π interaction in **3b** (interplanar distance of 3.415 Å) but not in **12c**: In this latter crystal structure, it contributes to the cohesion of the molecular columns through C–H···π hydrogen bonds (H···π = 2.83 Å) (Figure 2a). However, in **12c** the methylsulfanylphenyl groups are implied in the π–π stacking (interplanar distances of 3.261 Å and 3.549 Å). Such neighboring molecular columns interact through C–H···π,N,O,Cl (**3b**) or C–H···π,N,O,S (**12c**) hydrogen bonds and form infinite channels parallel to the [10] and [100] directions for **3b** and **12c**, respectively (Figure 2b). The free-diameter apertures of these channels are 5.39 Å × 3.52 Å and 4.87 Å × 2.64 Å for **3b** and **12c** respectively (i.e., van der Waals radii taken into account), large enough to accommodate *n*-hexane and chloroform molecules, which are disordered.

## Conclusion

In summary, we have described an efficient approach for the synthesis of uncommon 2,7-diazacarbazoles from readily available tetrahalogenated 4,4’-bipyridines. By use of the palladium-catalyzed double N-arylation of electron-rich anilines as the key reaction, the diazacarbazoles were regioselectively generated. Crystal structure determination shows that these molecules interact mainly through π–π stacking. The reported synthesis should widen the use of diazacarbazoles for biological and electronic applications; the easy insertion of substituents of different sizes is expected to influence the dominating molecular π–π stacking, which in turn may influence the solid-state properties of the prepared material [44–45].

## Experimental

All reactions were performed under an atmosphere of argon in oven-dried glassware. Toluene was distilled over sodium/benzophenone and stored over sodium. Melting points were measured on a Totoli apparatus. Proton and carbon NMR spectra were recorded on Bruker AMX-400, AC-200 or AC-250 Fourier transform spectrometers with an internal deuterium lock. Chemical shifts are quoted in parts per million (ppm) downfield of tetramethylsilane. Coupling constants *J* are quoted in Hz. Mass spectra with electronic impact (MS–EI) were recorded from a Shimadzu QP 2010 apparatus. High resolution mass spectra were recorded from a Bruker micrOTOFQ. All reagents were used as received. TLC was performed on silica gel plates and visualized with an UV lamp (254 nm). Chromatography was performed on silica gel (70–230 mesh).

**General procedure for the preparation of 3,6-dichloro-9-aryl-2,7-diazacarbazole 3a**–**g.** Argon was bubbled into a mixture of **2a** (100 mg, 0.26 mmol), amine **6** (0.234 mmol), Pd_2_(dba)_3_ (24 mg, 0.026 mmol), XPhos (37 mg, 0.078 mmol), and NaO*t*-Bu (75 mg, 0.78 mmol) in toluene (1.5 mL) for 15 min. The mixture was then heated at 120 °C in a sealed tube for 24 h. After cooling, the mixture was filtered through a pad of silica gel (dichloromethane/ethyl acetate 1/1). The filtrate was concentrated to give a residue, which was purified by column chromatography (silica gel: cyclohexane/ethyl acetate) to afford diazacarbazole **3**. The spectral and analytical data are given in Supporting Information File 1.

## Supporting Information

File 1 Characterization data and NMR spectra of all compounds, including X-ray structure determination of **3b** and **12c**.
