# Supplementary material for: Double N-arylation reaction of polyhalogenated 4,4’-bipyridines. Expedious synthesis of functionalized 2,7-diazacarbazoles
Source: Beilstein J Org Chem. 2012 Feb 14;8:253–8. doi: 10.3762/bjoc.8.26 (PMC3302087; doi:10.3762/bjoc.8.26)
Supplement: File 1 — Characterization data and NMR spectra of all compounds, including X-ray structure determination of 3b and 12c. [file Beilstein_J_Org_Chem-08-253-s001.pdf]

Supporting Information

for

Double N-arylation reaction of polyhalogenated 4,4'-bipyridines. Expedious synthesis of functionalized 2,7-diazacarbazoles

Mohamed Abboud[1,2], Emmanuel Aubert[2], Victor Mamane[1,*]

Address: [1]Structure et Réactivité des Systèmes Moléculaires Complexes (SRSMC), UMR CNRS-UHP 7565 Nancy Université, BP 70239, Bd des Aiguillettes, 54506 Vandoeuvre-les-Nancy, France and [2]Cristallographie, Résonance Magnétique et Modélisations (CRM2), UMR CNRS-UHP 7036 Nancy Université, BP 70239, Bd des Aiguillettes, 54506 Vandoeuvre-les-Nancy, France

Email: Victor Mamane* - victor.mamane@srsmc.uhp-nancy.fr

* Corresponding author

Characterization data and NMR spectra of all compounds, including X-ray structure determination of 3b and 12c.

Table of contents

1. Characterization of compounds **3** 2

2. Synthesis and characterization of compounds **9** 17

3. Synthesis and characterization of compound **10** 22

4. Synthesis and characterization of compounds **11** 24

5. Synthesis and characterization of compounds **12** 28

6. X-ray structure determination 34

1. Characterization of compounds 3

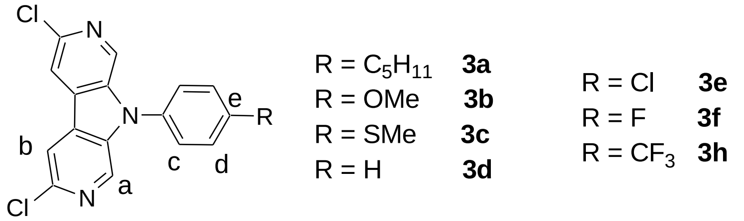

3,6-Dichloro-9-(4-pentylphenyl)-2,7-diazacarbazole (3a). Mp 128–130 °C; ^{1}H NMR (250 MHz, $CDCl_3$) δ 8.65 (s, 2H, H_a), 8.00 (s, 2H, H_b), 7.45 (s, 4H, $H_{c,d}$), 2.75 (t, J = 7.5 Hz, 2H, CH_2), 1.73 (quint, J = 7.5 Hz, 2H, CH_2), 1.39 (m, 4H, CH_2), 0.93 (t, J = 7 Hz, 3H, CH_3); ^{13}C NMR (63 MHz, $CDCl_3$) δ 144.3, 141.6, 137.3, 133.9, 132.6, 130.5, 129.1, 126.1, 115.8, 35.7, 31.2, 31.0, 22.5, 14.0; MS (70 eV) m/z (%): 383 (95, M^+), 326 (100), 290 (15); HRMS m/z: calcd for $C_{21}H_{20}Cl_2N_3$ $(M + H)^+$ 384.1029, found: 384.1058.

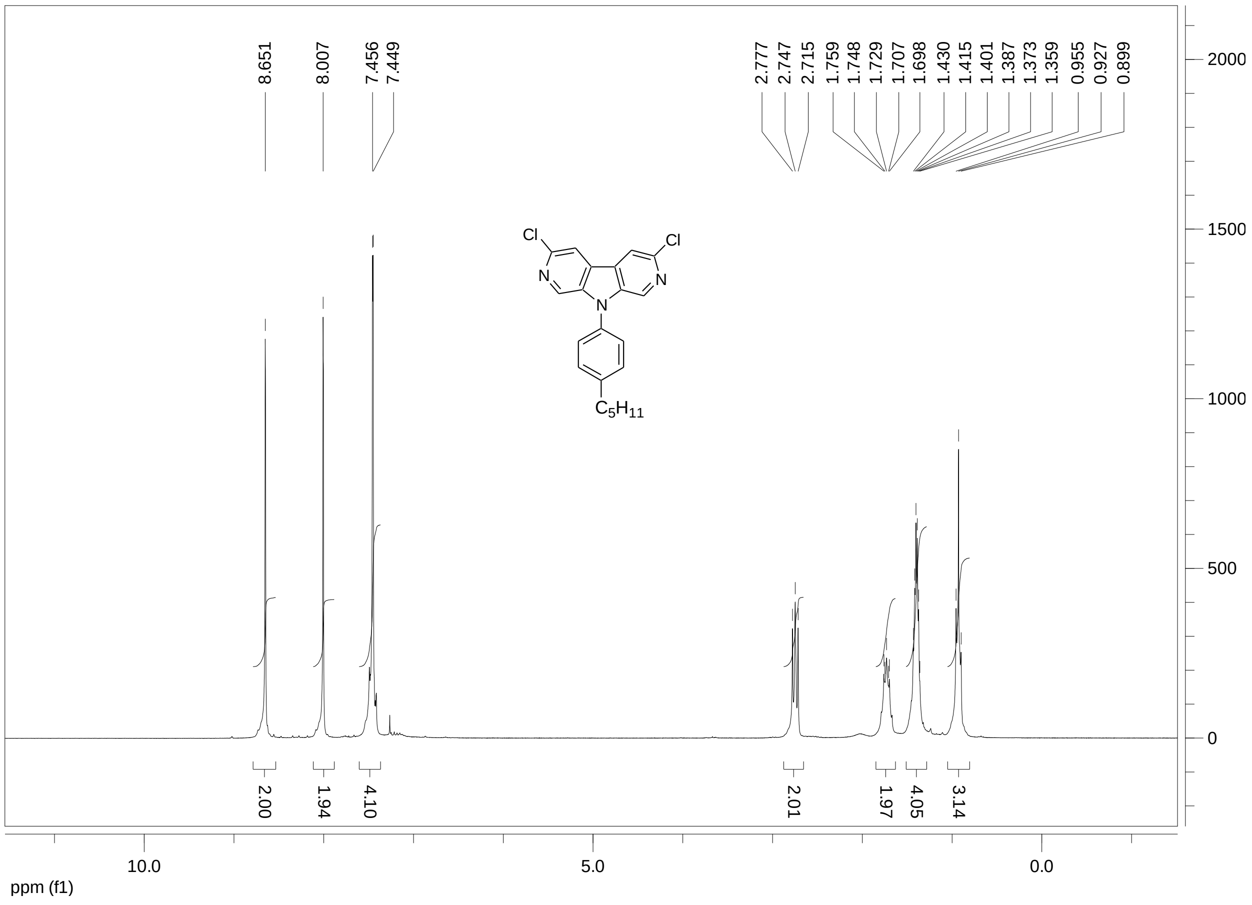

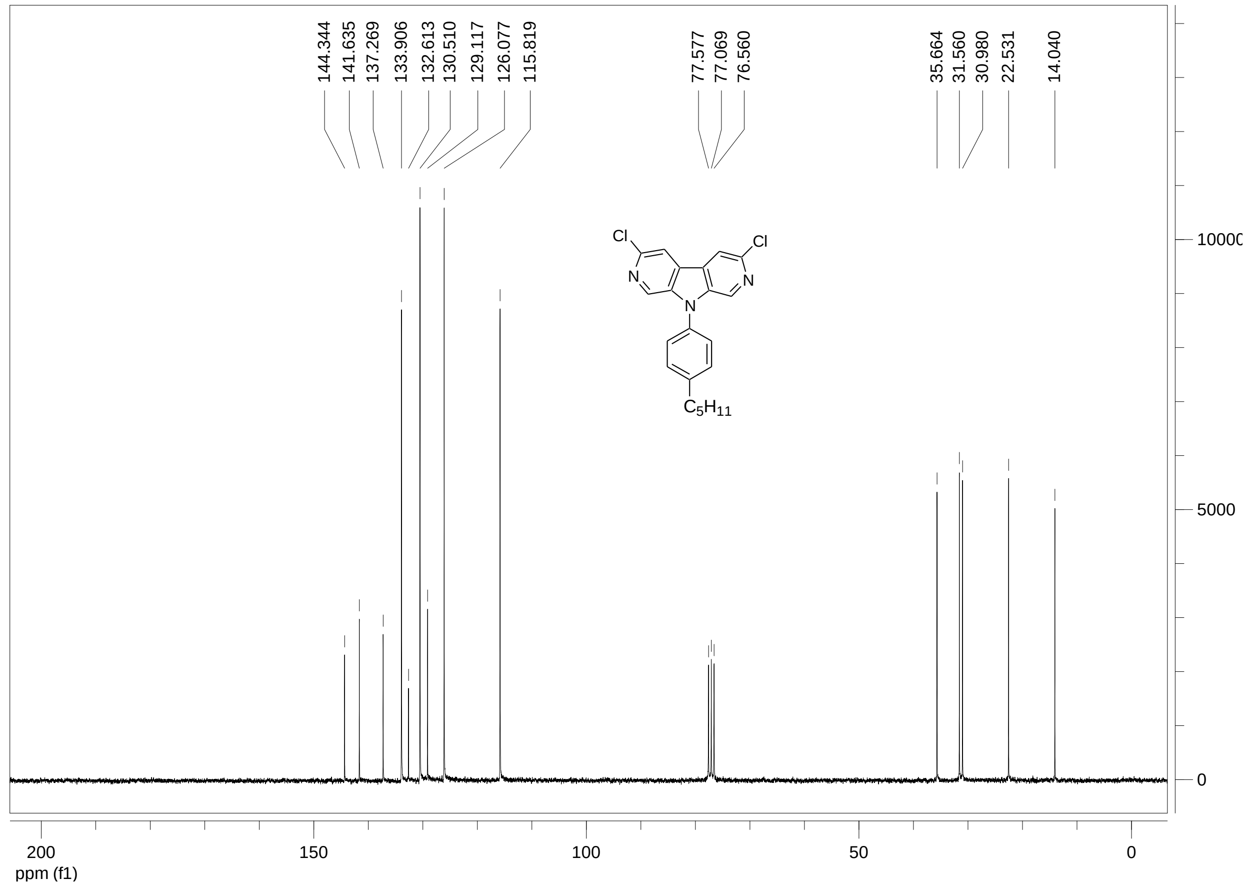
144.344
141.635
137.269
133.906
132.613
130.510
129.117
126.077
115.819
77.577
77.069
76.560
35.664
31.560
30.980
22.531
14.040
Cl
Cl
N
N
N
C5H11
10000
5000
0
200
150
100
50
0
ppm (f1)

3,6-Dichloro-9-(4-methoxyphenyl)-2,7-diazacarbazole (3b). Mp 178–180 °C; ^{1}H NMR (200 MHz, $CDCl_3$) δ 8.61 (s, 2H, H_a), 8.03 (s, 2H, H_b), 7.47 (d, J = 8.8 Hz, 2H, H_c), 7.17 (d, J = 8.8 Hz, 2H, H_d), 3.95 (s, 3H, OCH_3); ^{13}C NMR (50 MHz, $CDCl_3$) δ 160.3, 141.9, 137.9, 134.1, 129.3, 128.1, 127.8, 116.1, 116.05, 55.0; MS (70 eV) m/z (%): 343 (100, M^+), 328 (40), 300 (10), 264 (15); HRMS m/z: calcd for $C_{17}H_{12}Cl_2N_3O$ $(M + H)^+$ 344.0352, found: 344.0365.

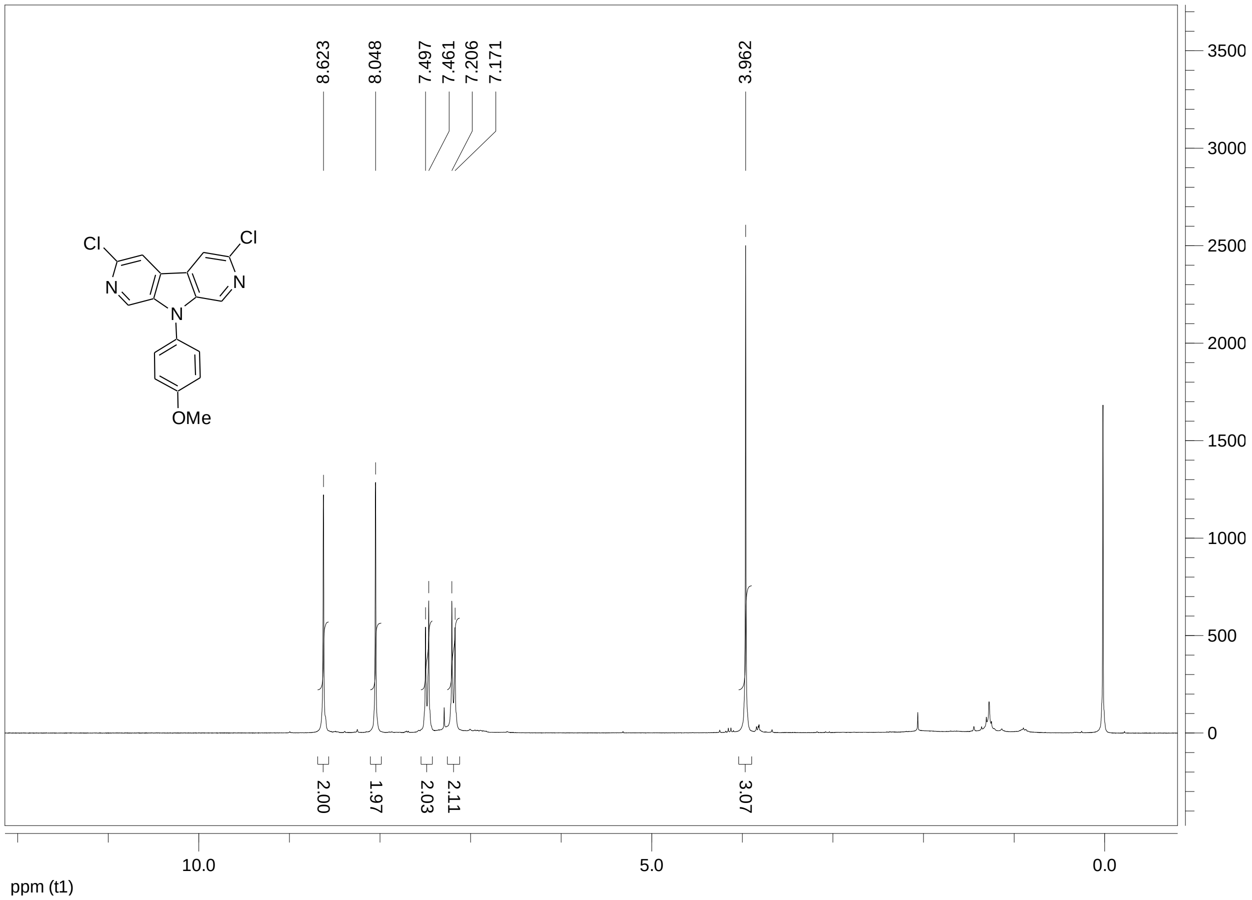

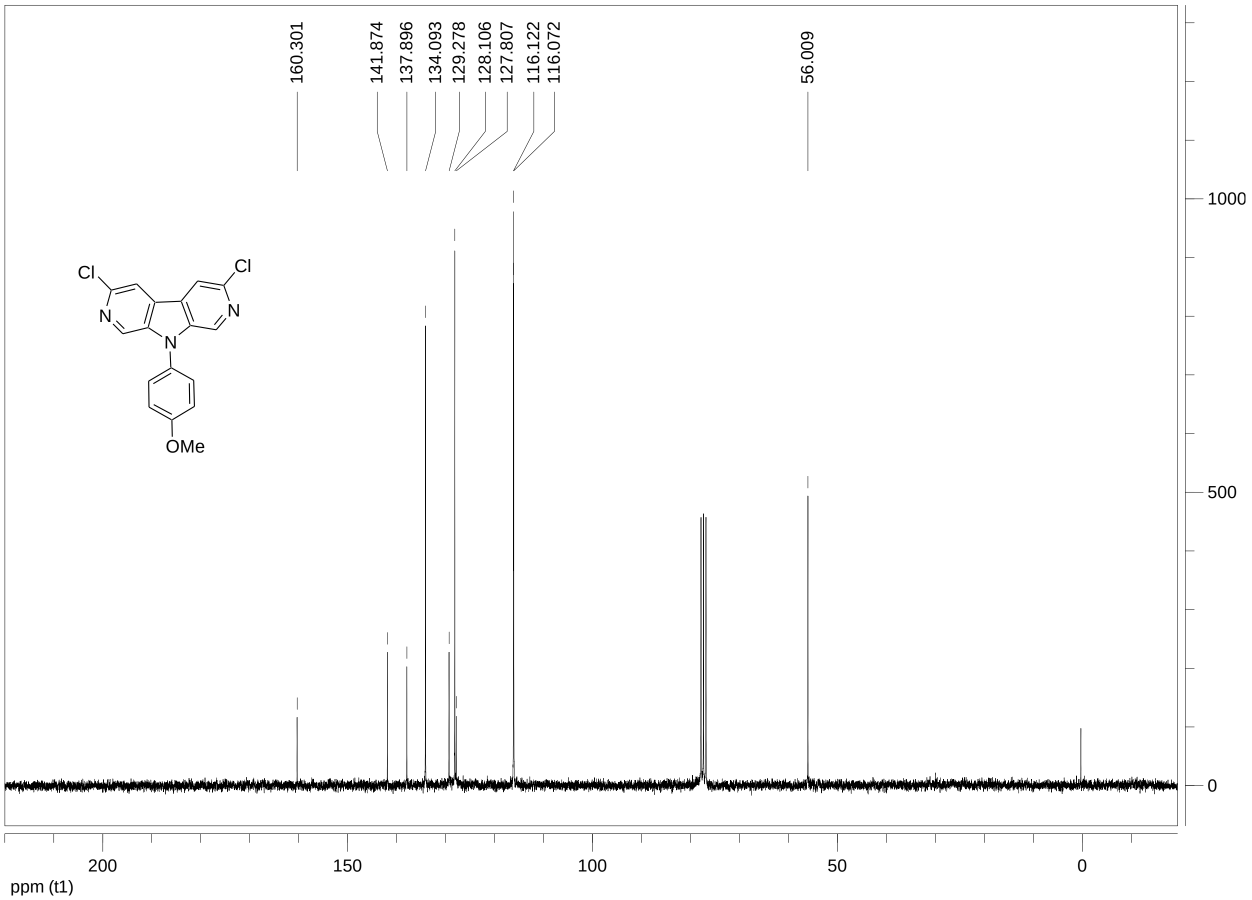
160.301
141.874
137.896
134.093
129.278
128.106
127.807
116.122
116.072
56.009
Cl
Cl
N
N
N
OMe
1000
500
0
200
150
100
50
0
ppm (t1)

3,6-Dichloro-9-(4-thiomethylphenyl)-2,7-diazacarbazole (3c). Mp 196–198 °C; ^{1}H NMR (250 MHz, $CDCl_3$) δ 8.65 (d, J = 1 Hz, 2H, H_a), 8.03 (d, J = 1 Hz, 2H, H_b), 7.46 and 7.52 (2d, AB syst., J = 9 Hz, 4H, $H_{c,d}$), 2.61 (s, 3H, SCH_3); ^{13}C NMR (63 MHz, $CDCl_3$) δ 141.7, 140.6, 137.1, 133.6, 131.6, 129.1, 127.7, 126.6, 115.8, 15.4; MS (70 eV) m/z (%): 359 (100, M^+), 344 (45), 108 (12); HRMS m/z: calcd for $C_{17}H_{12}Cl_2N_3S$ $(M + H)^+$ 360.0123, found: 360.0144.

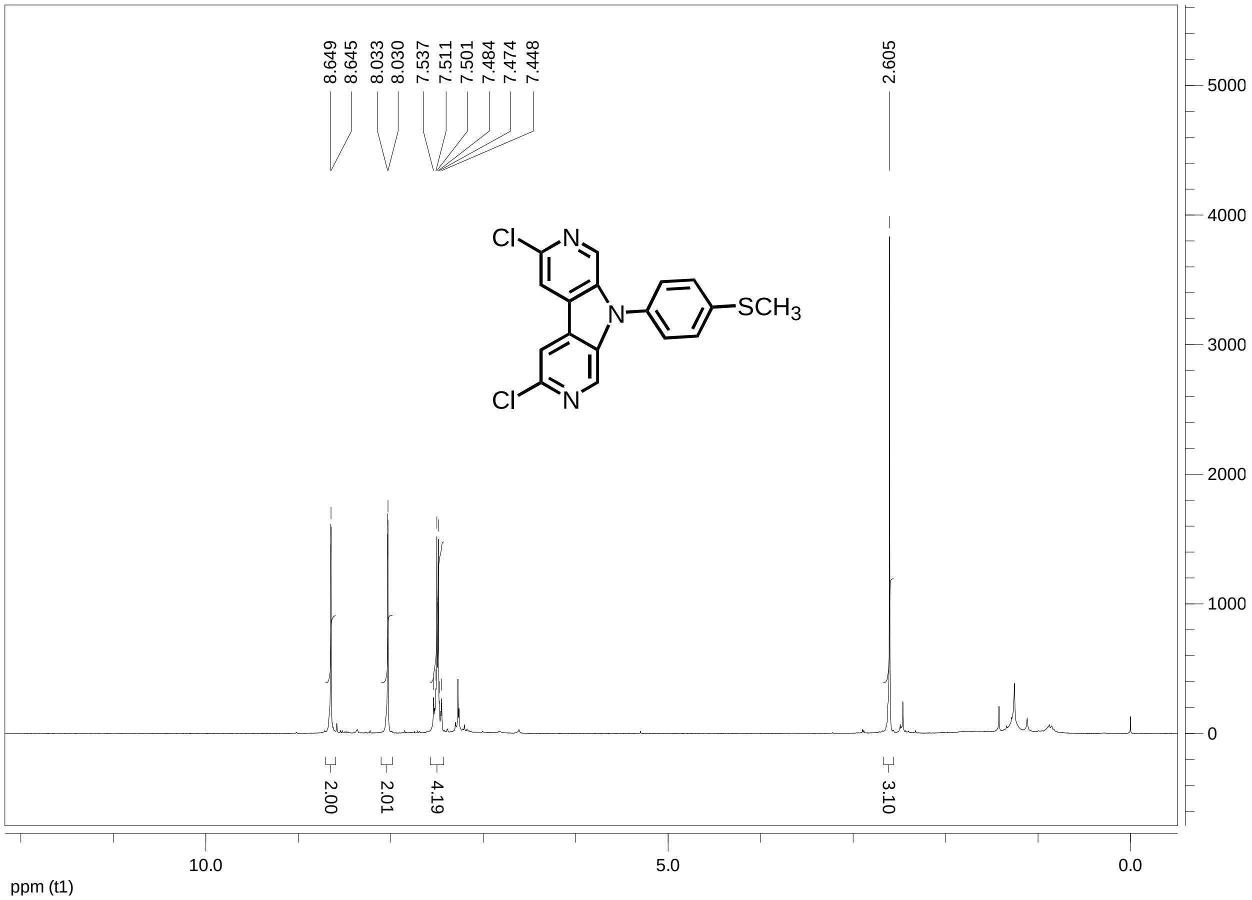

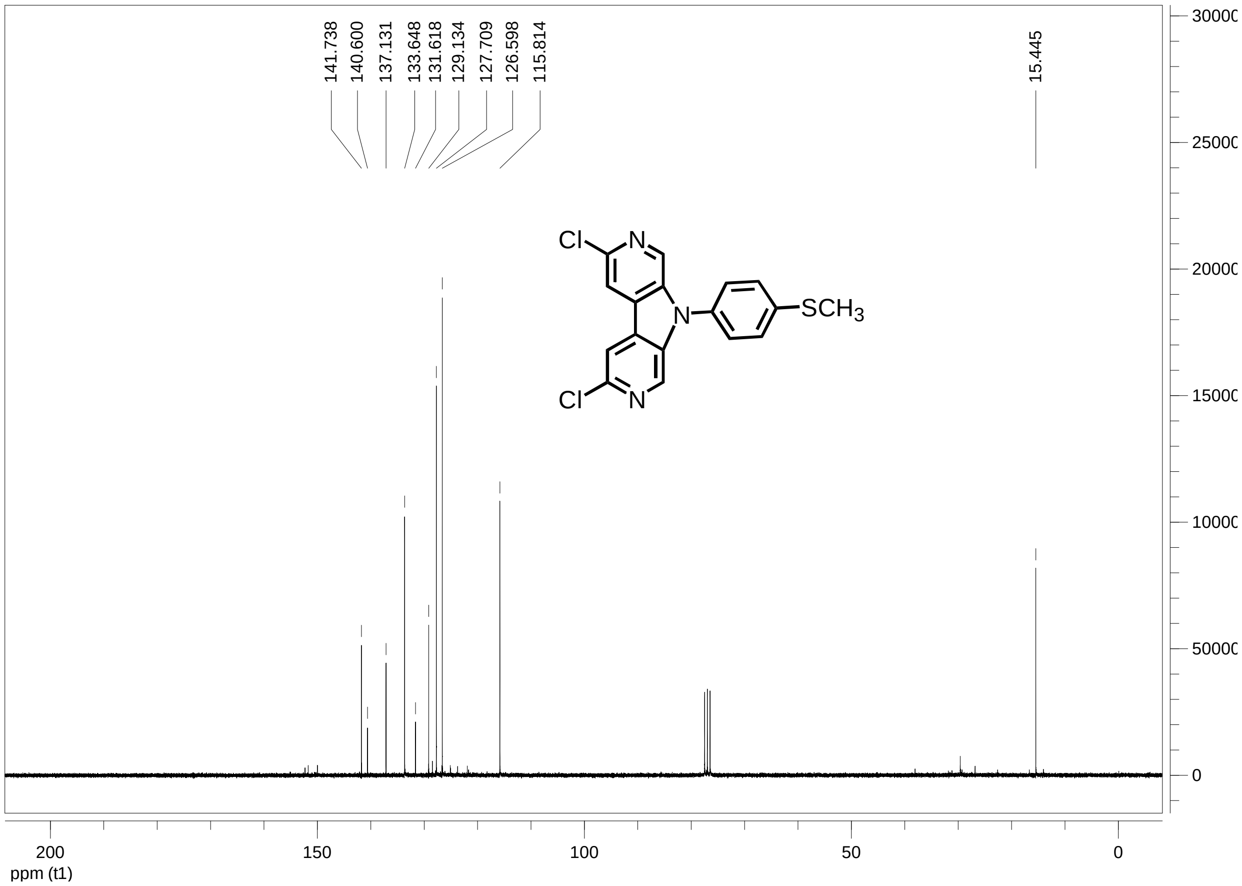

141.738
140.600
137.131
133.648
131.618
129.134
127.709
126.598
115.814
15.445
Cl
N
N
SCH3
Cl
N
30000
25000
20000
15000
10000
50000
0
200
150
100
50
0
ppm (t1)

3,6-Dichloro-9-phenyl-2,7-diazacarbazole (3d). Mp 227–229 °C; ^{1}H NMR (250 MHz, $CDCl_3$) δ 8.69 (d, *J* = 1 Hz, 2H, H_a), 8.05 (d, *J* = 1 Hz, 2H, H_b), 7.97 (d, *J* = 7.5 Hz, 2H, H_c), 7.59 (dt, *J* = 7.5, 1 Hz, 3H, $H_{d,e}$); ^{13}C NMR (63 MHz, $CDCl_3$) δ 141.9, 137.2, 135.2, 133.8, 130.7, 129.3, 129.2, 126.3, 115.9; MS (70 eV) m/z (%): 313 (100, M^+), 277 (10), 243 (12), 77 (18), 51 (17); HRMS m/z: calcd for $C_{16}H_{10}Cl_2N_3$ $(M + H)^+$ 314.0246, found: 314.0257.

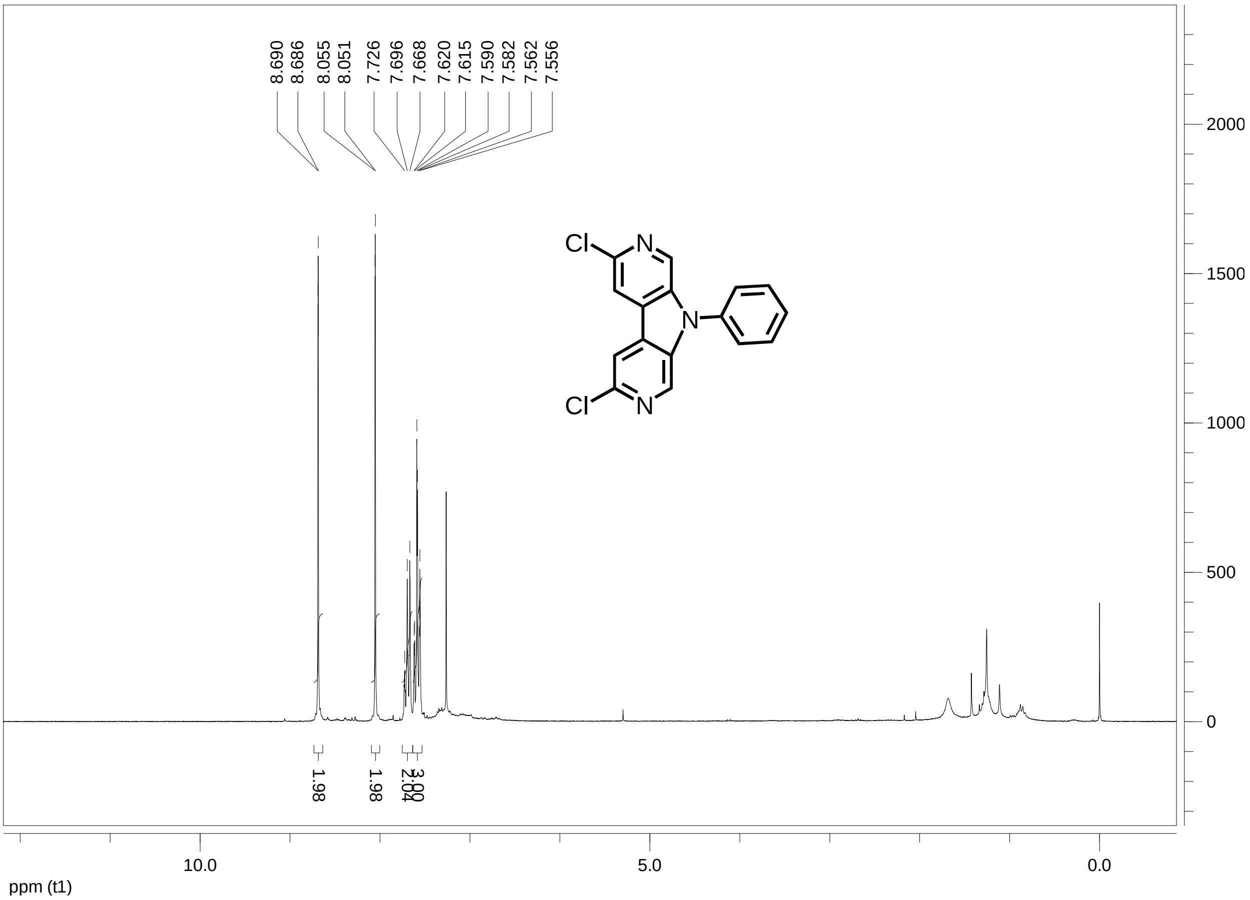

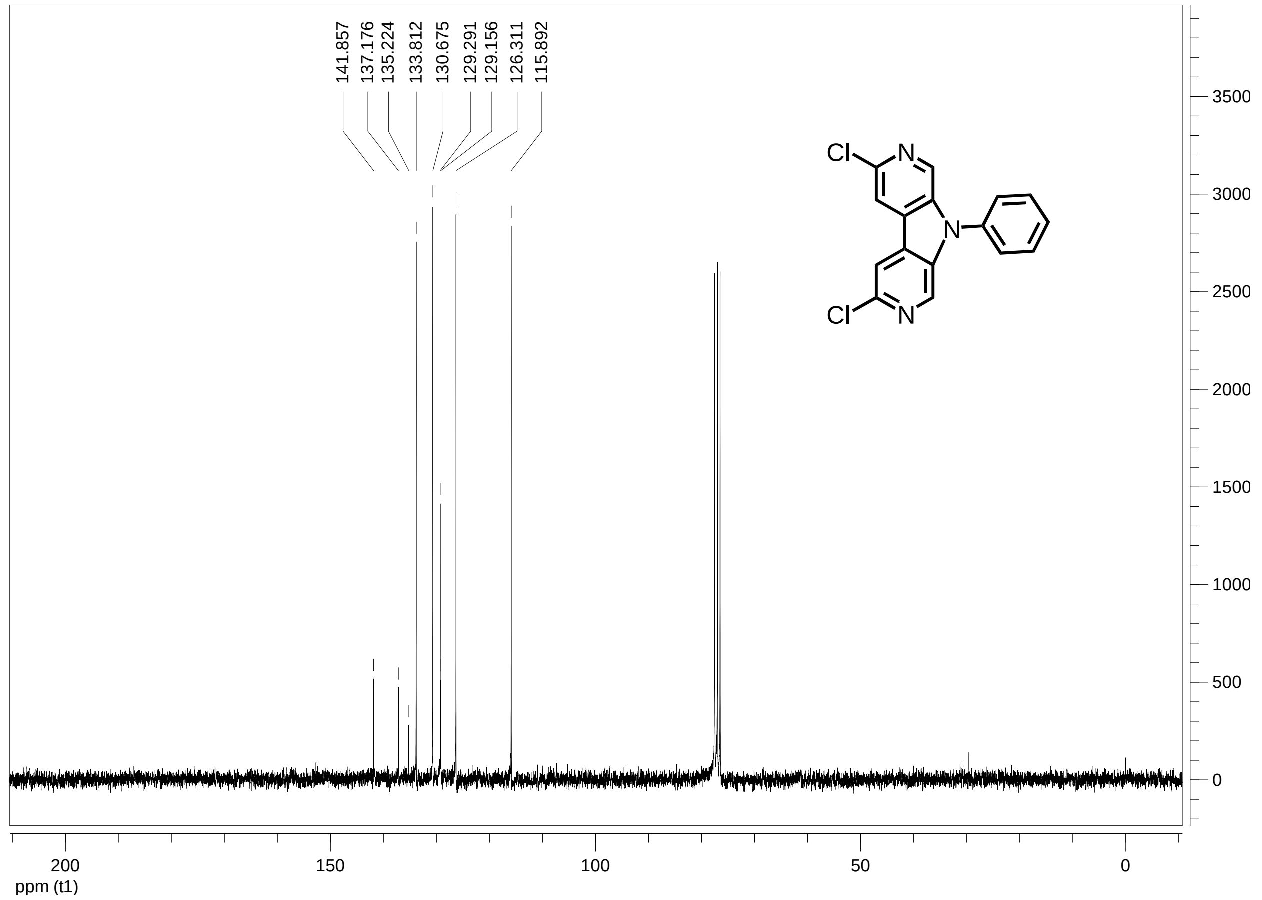
141.857
137.176
135.224
133.812
130.675
129.291
129.156
126.311
115.892
Cl
N
N
Cl
N
3500
3000
2500
2000
1500
1000
500
0
200
150
100
50
0
ppm (t1)

3,6-Dichloro-9-(4-chlorophenyl)-2,7-diazacarbazole (3e). Mp > 250 °C; ^{1}H NMR (250 MHz, d_6-DMSO) δ 8.66 (s, 2H, H_a), 8.06 (s, 2H, H_b), 7.68 (d, J = 8.7 Hz, 2H, H_c), 7.53 (d, J = 8.7 Hz, 2H, H_d); ^{13}C NMR (63 MHz, d_6-DMSO) δ 141.2, 137.5, 134.8, 134.5, 133.8, 131.2, 129.9, 129.1, 117.4; MS (70 eV) m/z (%): 347 (100), 277 (25), 241 (15), 75 (20); HRMS m/z: calcd for $C_{16}H_9Cl_3N_3$ $(M + H)^+$ 347.9857, found: 347.9882.

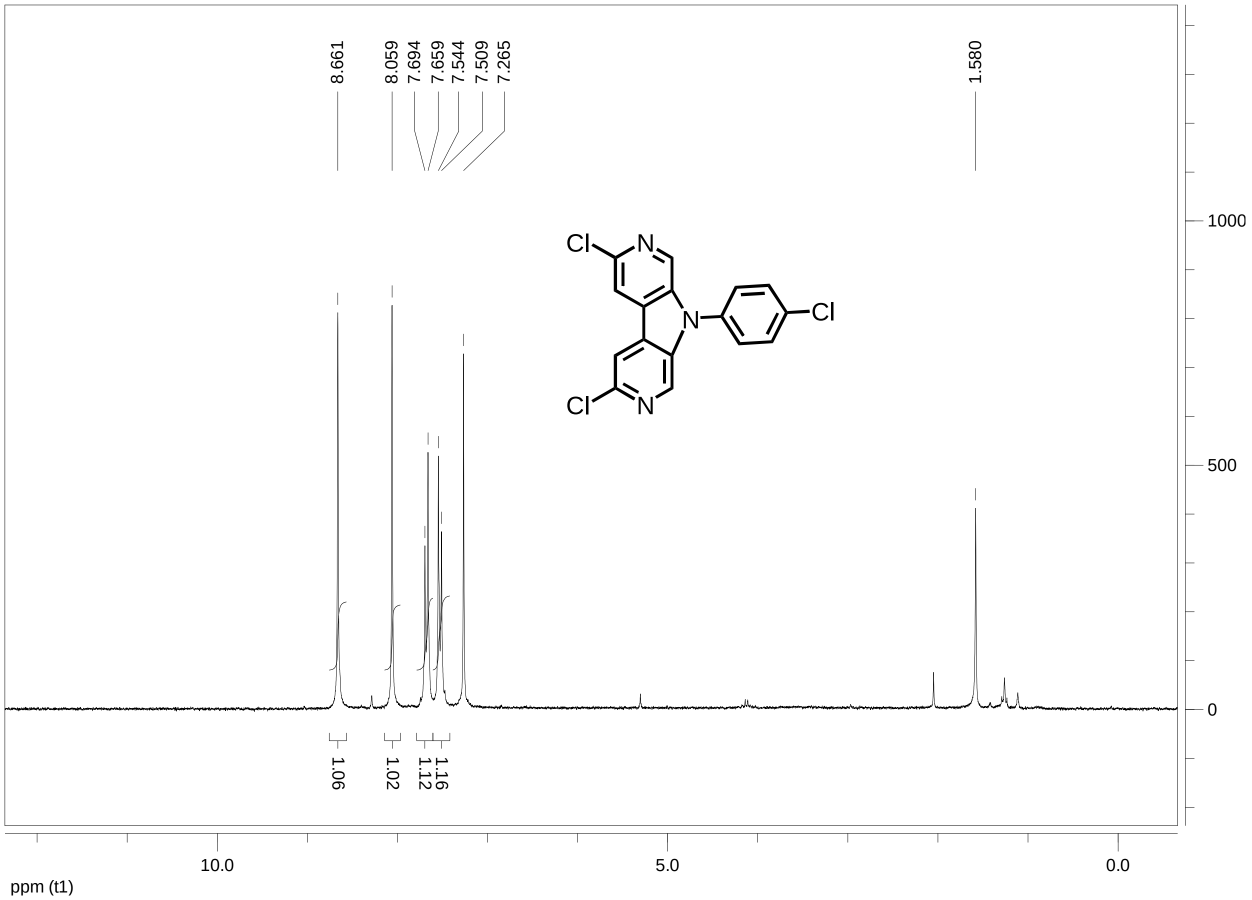

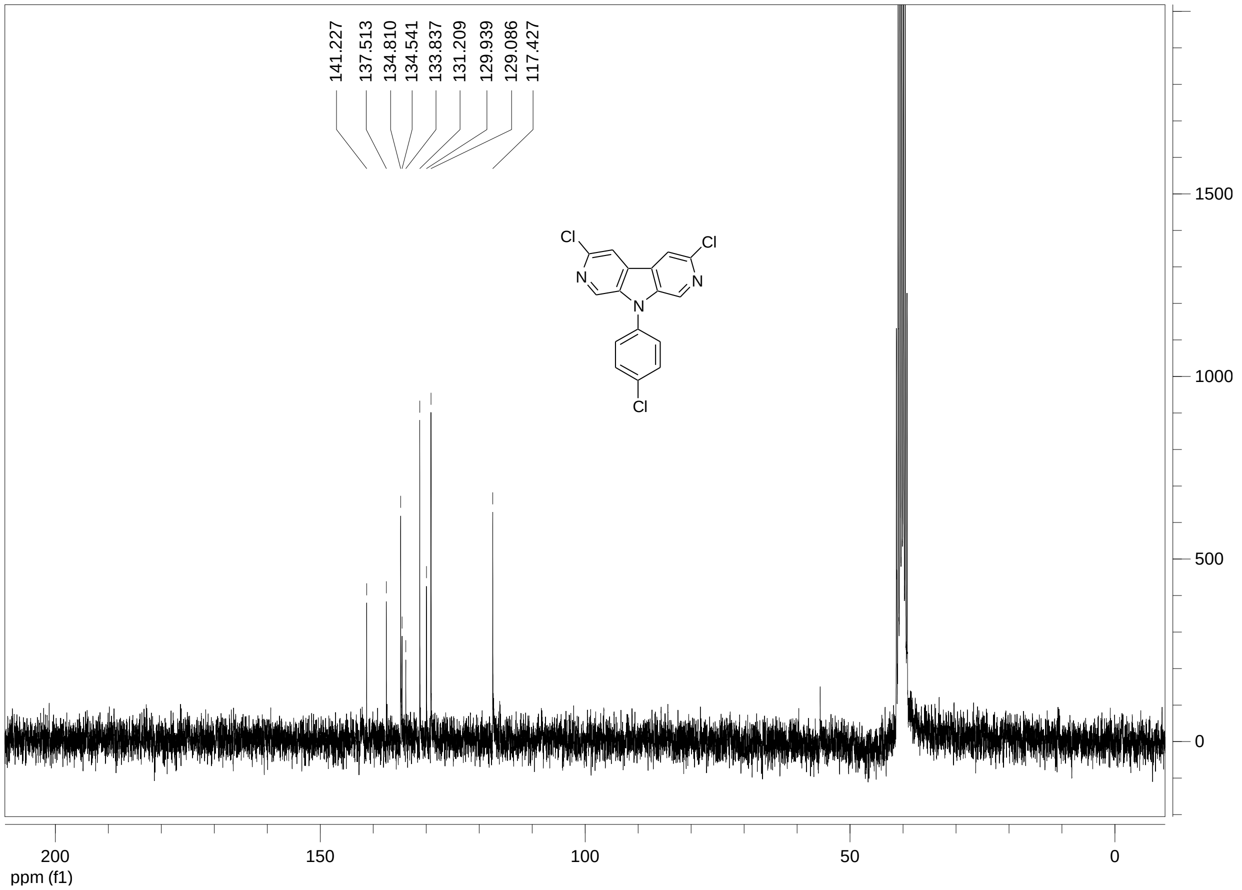
141.227
137.513
134.810
134.541
133.837
131.209
129.939
129.086
117.427
Cl
Cl
N
N
N
Cl
1500
1000
500
0
200
150
100
50
0
ppm (f1)

3,6-Dichloro-9-(4-fluorophenyl)-2,7-diazacarbazole (3f). Mp > 250 °C; ^{1}H NMR (250 MHz, d_6-DMSO) δ 8.72 (d, J = 1 Hz, 2H, H_a), 8.61 (d, J = 1 Hz, 2H, H_b), 7.88 (dd, J = 9, 5 Hz, 2H, H_c), 7.57 (t, J = 9 Hz, 2H, H_d); ^{13}C NMR (63 MHz, d_6-DMSO) δ 140.4, 137.1, 134.0, 131.1, 129.0, 128.9, 117.6, 117.2, 116.7; MS (70 eV) m/z (%): 331 (100, M^+), 261 (12), 233 (10), 75 (12); HRMS m/z: calcd for $C_{16}H_9Cl_2FN_3$ $(M + H)^+$ 332.0152, found: 332.0161.

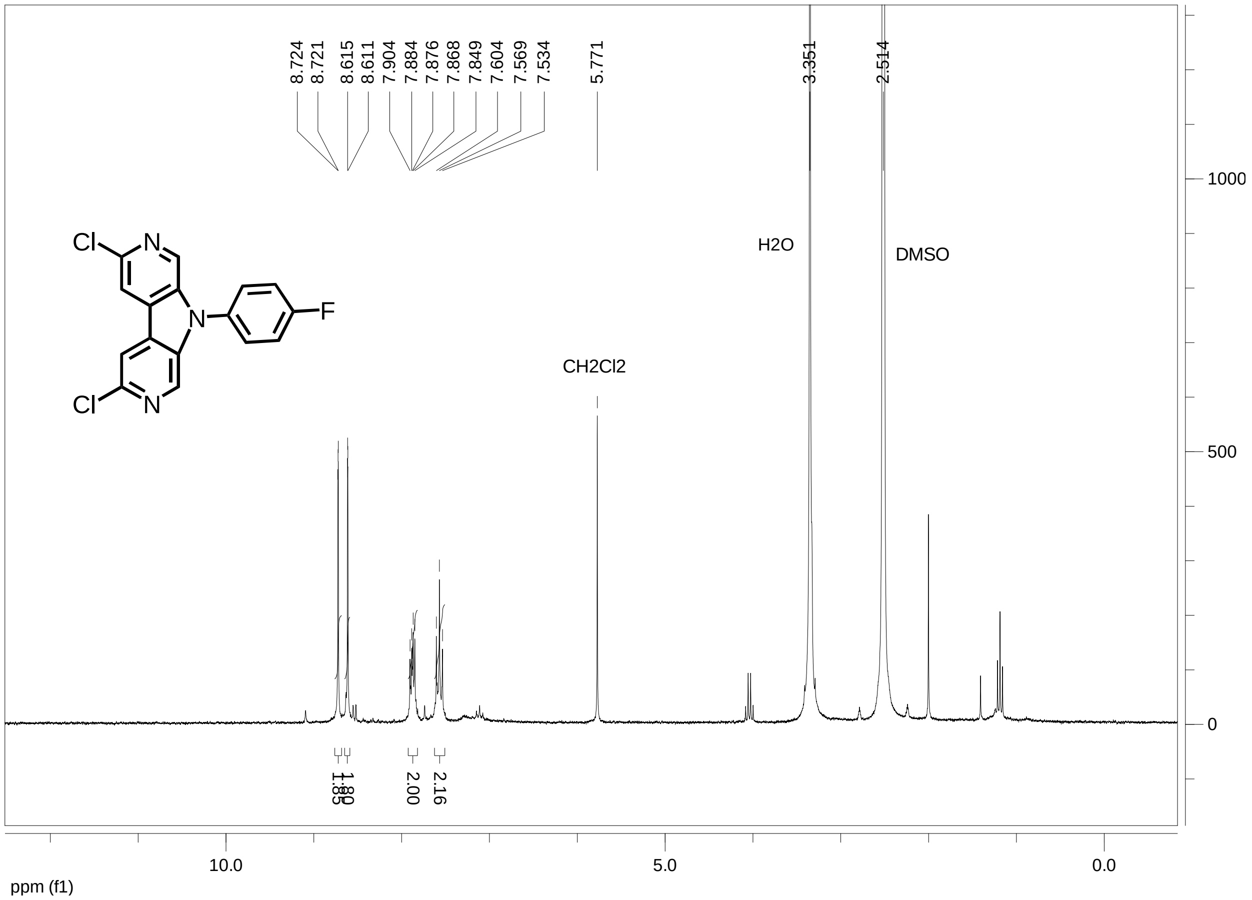

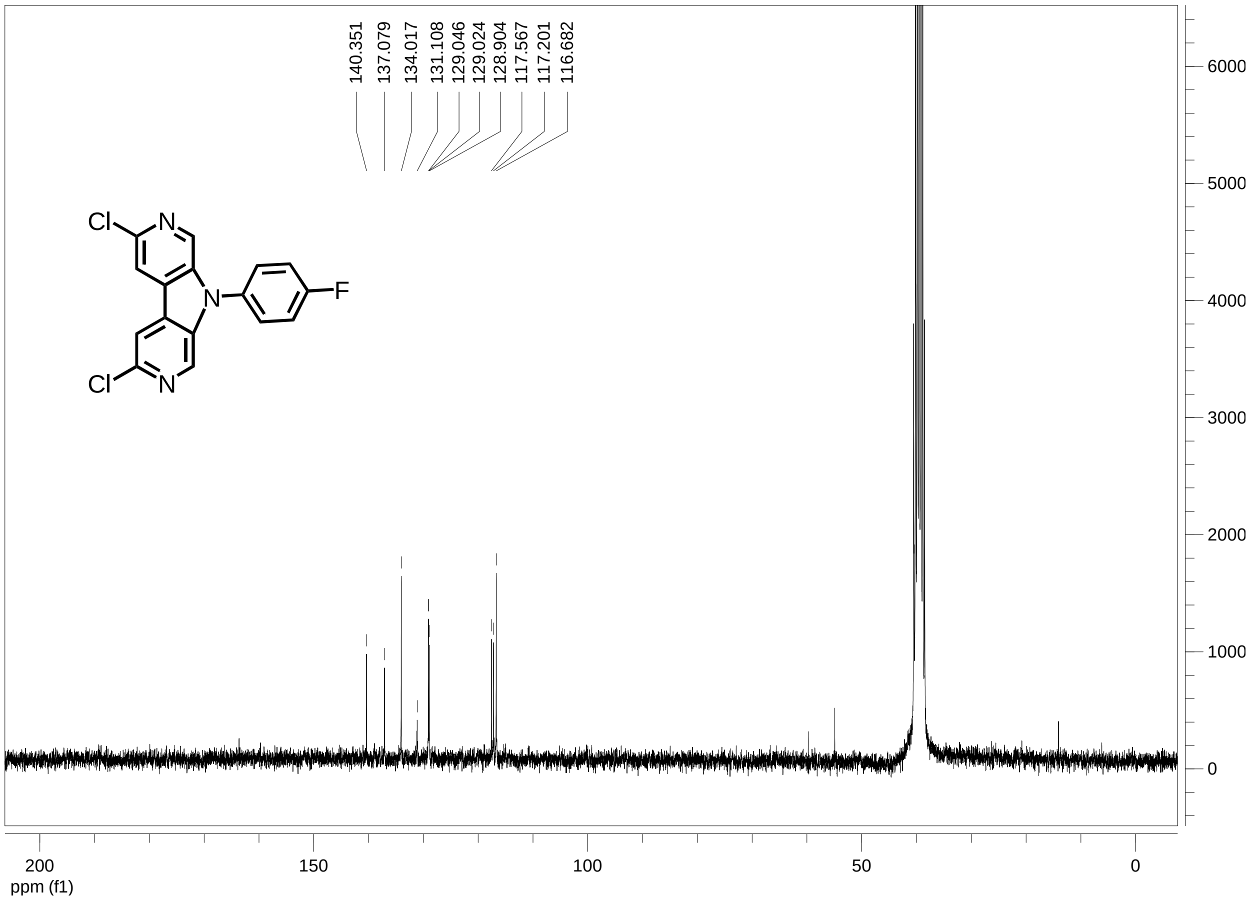
140.351
137.079
134.017
131.108
129.046
129.024
128.904
117.567
117.201
116.682
Cl
N
N
F
Cl
N
6000
5000
4000
3000
2000
1000
0
200
150
100
50
0
ppm (f1)

3,6-Dichloro-9-(4-trifluoromethylphenyl)-2,7-diazacarbazole (3g). Mp > 250 °C; ^{1}H NMR (250 MHz, d_6-DMSO) δ 8.83 (d, J = 0.8 Hz, 2H, H_a), 8.59 (d, J = 0.8 Hz, 2H, H_b), 8.06 (s, 4H, $H_{c,d}$); ^{13}C NMR (63 MHz, d_6-DMSO) δ 141.5, 139.4, 137.2, 134.9, 130.3, 129.0 (t, J = 38.6 Hz), 128.3 (d, J = 4.3 Hz), 127.8, 122.5 (t, J = 325 Hz), 117.5; MS (70 eV) m/z (%): 381 (100, M^+), 277 (12); HRMS m/z: calcd for $C_{17}H_9Cl_2F_3N_3$ $(M + H)^+$ 382.0120, found: 382.0127.

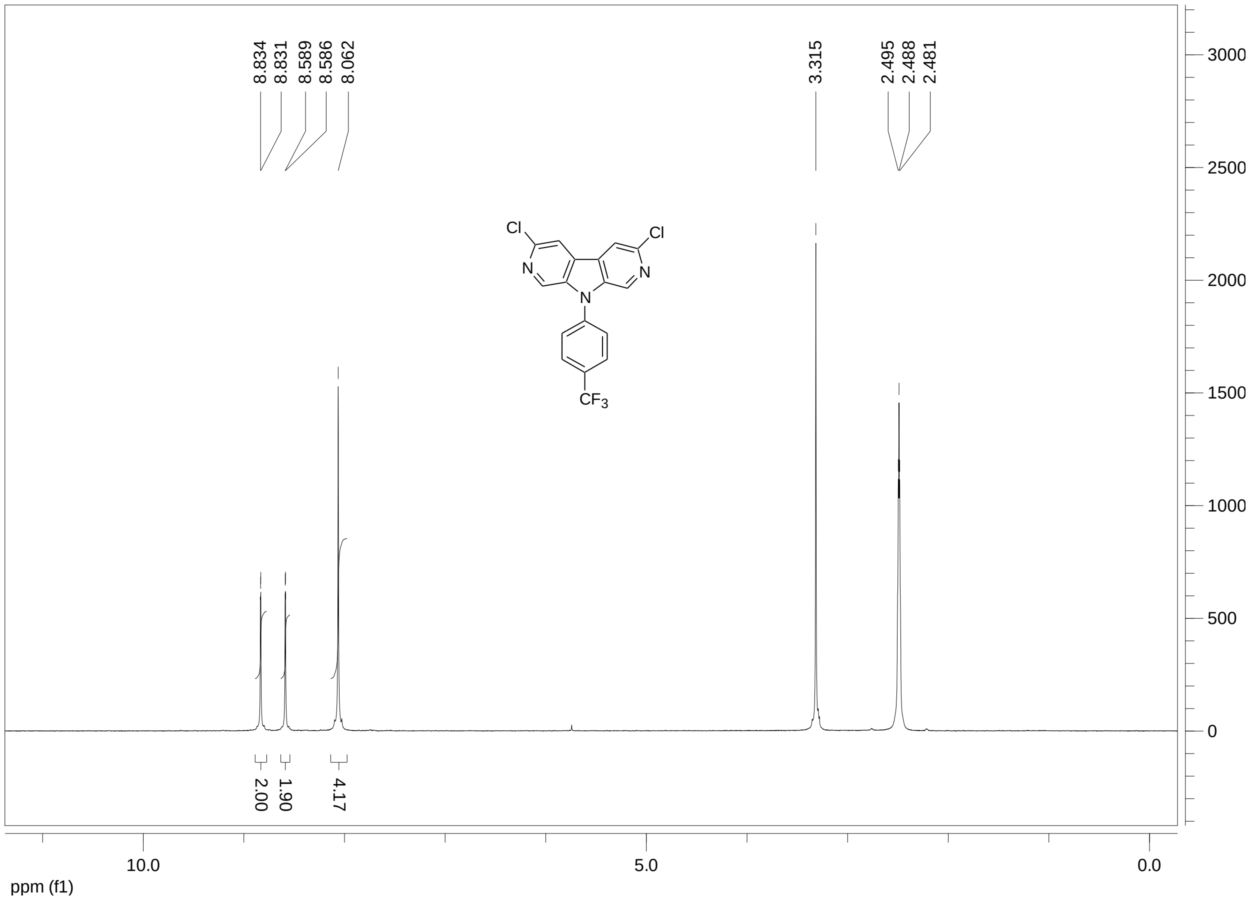

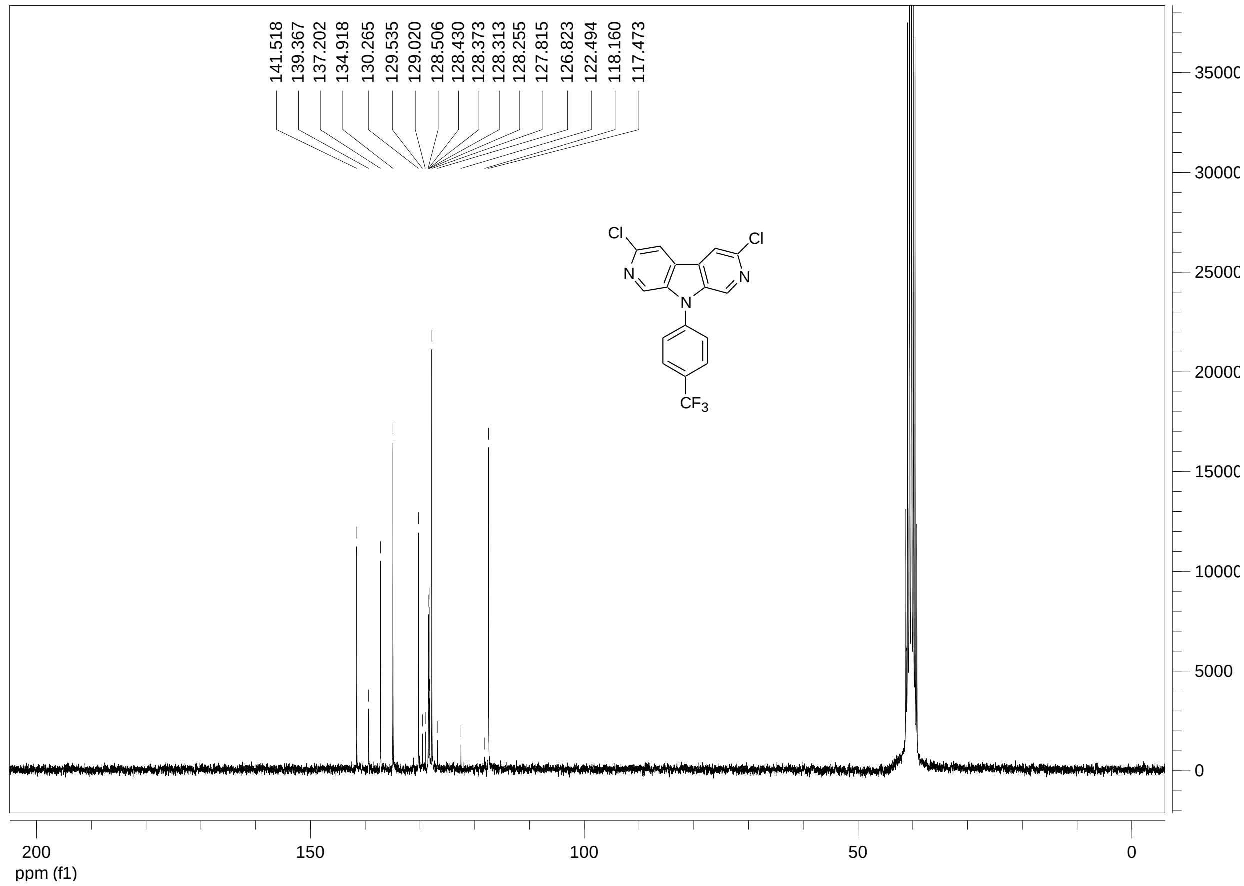
141.518
139.367
137.202
134.918
130.265
129.535
129.020
128.506
128.430
128.373
128.313
128.255
127.815
126.823
122.494
118.160
117.473
Cl
Cl
N
N
N
CF3
35000
30000
25000
20000
15000
10000
5000
0
200
150
100
50
0
ppm (f1)

2. Synthesis and characterization of compounds 9.

To a mixture of **2a** (50 mg, 0.13 mmol), **8** (144 mg, 0.39 mmol) and $PdCl_2(PPh_3)_2$ (9.1 mg, 0.013 mmol) under argon, degassed toluene (1.5 mL) was added. After heating at 100 °C for 18 h, the mixture was cooled to room temperature. Water was added and the mixture was extracted with diethyl ether, washed with brine and concentrated. The residue was purified by chromatography on silica gel (cyclohexane/ethyl acetate/triethylamine 9:0.5:0.5) to give separately **9a** (22 mg, 40%) and **9b** (20 mg, 33%).

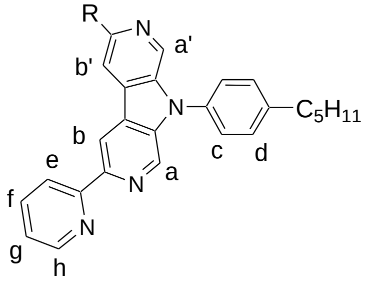

R = Cl **9a**
R = 2-pyridine **9b**

3-Chloro-6-(2-pyridyl)-9-(4-pentylphenyl)-2,7-diazacarbazole (9a). ^{1}H NMR (250 MHz, $CDCl_3$) δ 9.16 (s, 1H, H_a), 8.97 (s, 1H, $H_{a'}$), 8.72 (d, *J* = 5 Hz, 1H, H_h), 8.68 (s, 1H, H_b), 8.45 (d, *J* = 7.5 Hz, 1H, H_e), 8.14 (s, 1H, $H_{b'}$), 7.83 (t, *J* = 7.5 Hz, 1H, H_f), 7.50 (m, 4 H, $H_{c,d}$), 7.28 (dd, *J* = 7.5, 5 Hz, 1H, H_g), 2.77 (t, *J* = 7.5 Hz, 2H, CH_2), 1.75 (quint, *J* = 7.5 Hz, 2H, CH_2), 1.42 (m, 4H, CH_2), 0.95 (m, 3H, CH_3); ^{13}C NMR (63 MHz, $CDCl_3$) δ 156.2, 149.1, 147.8, 144.0, 141.6, 138.2, 137.1, 137.0, 133.8, 133.6, 133.0, 131.0, 130.4, 127.3, 126.1, 123.1, 120.9, 116.0, 113.3, 35.7, 31.5, 31.0, 22.5, 14.0; MS (70 eV) m/z (%): 426 (75, M^+), 369 (42), 167 (35); HRMS m/z: calcd for $C_{26}H_{24}ClN_4$ $(M + H)^+$ 427.1684, found: 427.1716.

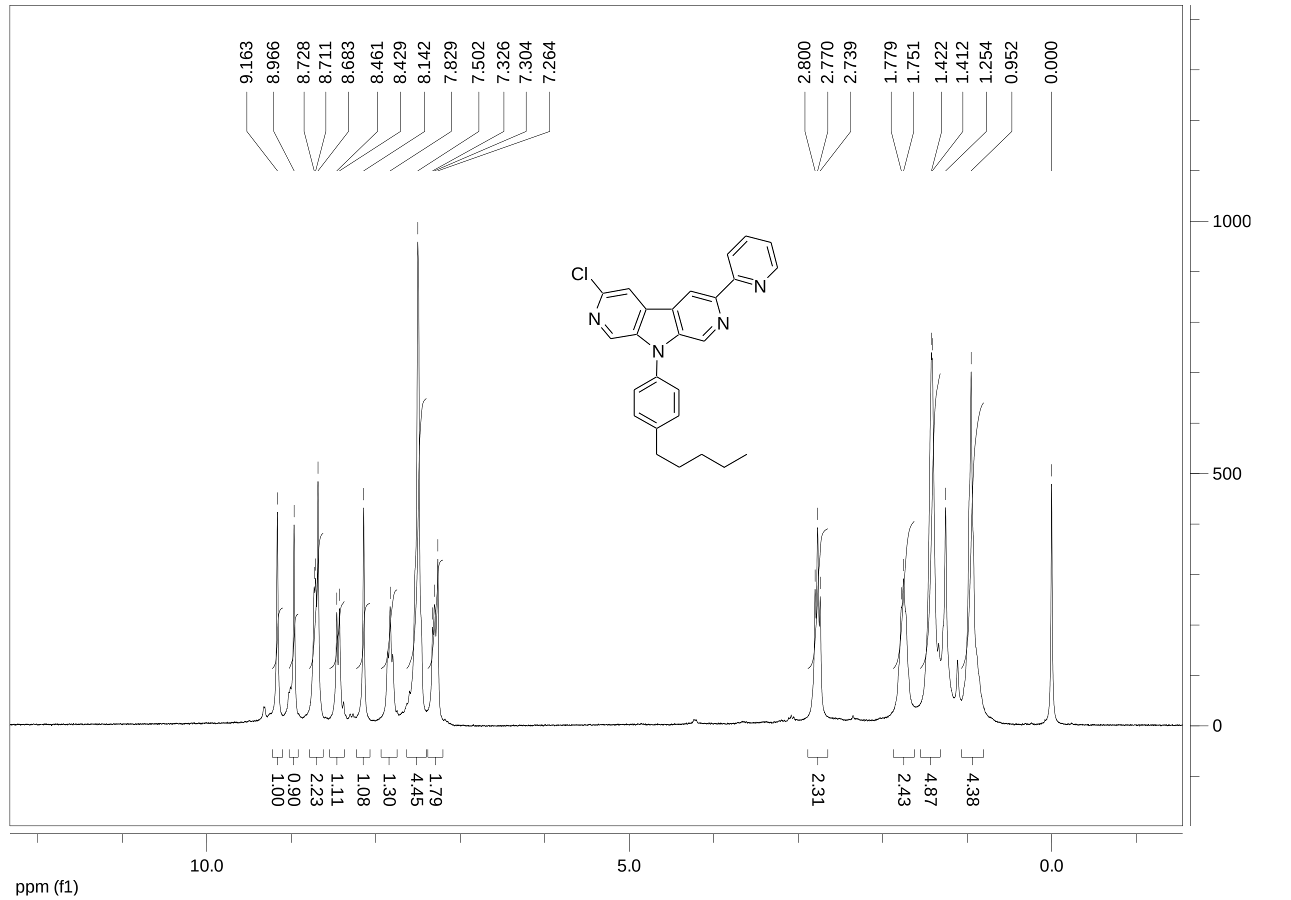
9.163
8.966
8.728
8.711
8.683
8.461
8.429
8.142
7.829
7.502
7.326
7.304
7.264
2.800
2.770
2.739
1.779
1.751
1.422
1.412
1.254
0.952
0.000
1000
500
0
1.00
0.90
2.23
1.11
1.08
1.30
4.45
1.79
2.31
2.43
4.87
4.38
10.0
5.0
0.0
ppm (f1)
Cl
N
N
N
N

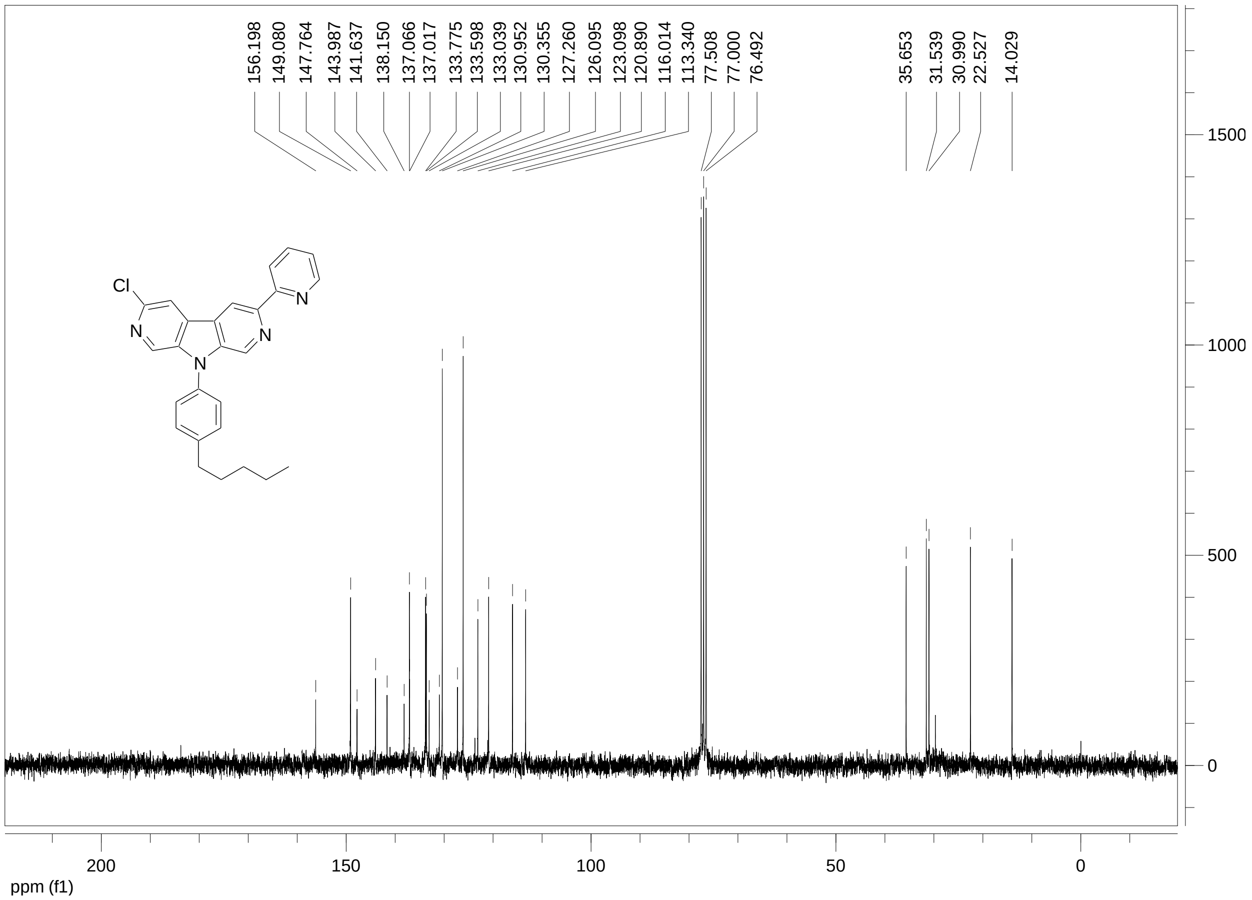
156.198
149.080
147.764
143.987
141.637
138.150
137.066
137.017
133.775
133.598
133.039
130.952
130.355
127.260
126.095
123.098
120.890
116.014
113.340
77.508
77.000
76.492
35.653
31.539
30.990
22.527
14.029
Cl
N
N
N
N
1500
1000
500
0
200
150
100
50
0
ppm (f1)

3,6-Bis(2-pyridyl)-9-(4-pentylphenyl)-2,7-diazacarbazole (9b). Mp 148–150 °C; ^{1}H NMR (250 MHz, $CDCl_3$) δ 9.32 (s, 2H, H_a), 9.01 (s, 2H, H_b), 8.74 (d, *J* = 4.8 Hz, 2H, H_h), 8.44 (d, *J* = 7.5 Hz, 2H, H_e), 7.82 (t, *J* = 7.5 Hz, 2H, H_f), 7.59 (d, *J* = 8.3 Hz, 2H, H_c), 7.49 (d, *J* = 8.3 Hz, 2H, H_d), 7.28 (dd, *J* = 7.5, 4.8 Hz, 2H, H_g), 2.78 (t, *J* = 7.5 Hz, 2H, CH_2), 1.75 (quint, *J* = 7.5 Hz, 2H, CH_2), 1.42 (m, 4H, CH_2), 0.96 (t, *J* = 5.5 Hz, 3H, CH_3); ^{13}C NMR (63 MHz, $CDCl_3$) δ 156.5, 149.2, 147.9, 143.6, 137.9, 136.9, 133.6, 133.5, 130.2, 129.0, 126.1, 122.9, 120.8, 113.7, 35.7, 31.5, 31.0, 22.5, 14.1; MS (70 eV) m/z (%): 469 (100, M^+), 412 (98); HRMS m/z: calcd for $C_{31}H_{28}N_5$ $(M + H)^+$ 470.2339, found: 470.2359.

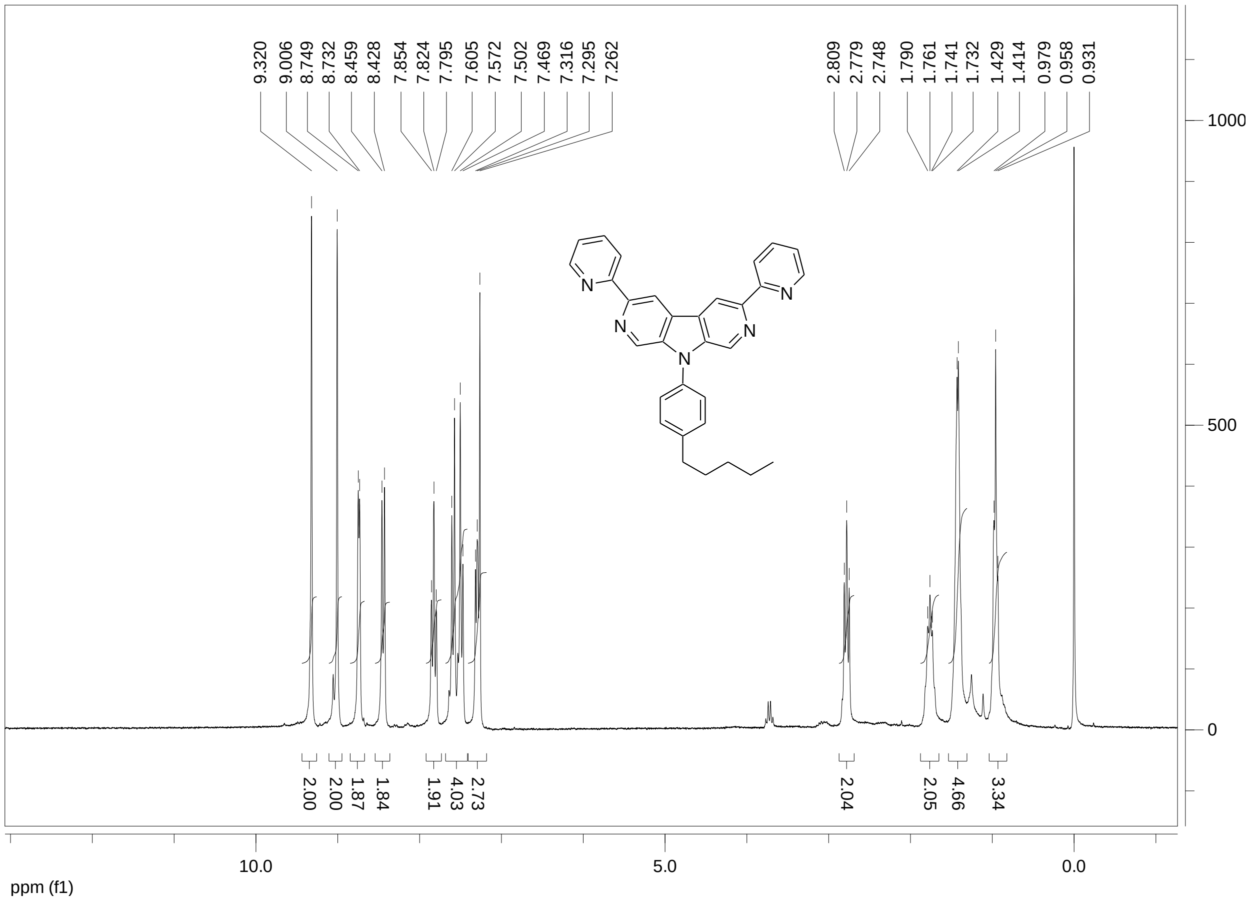

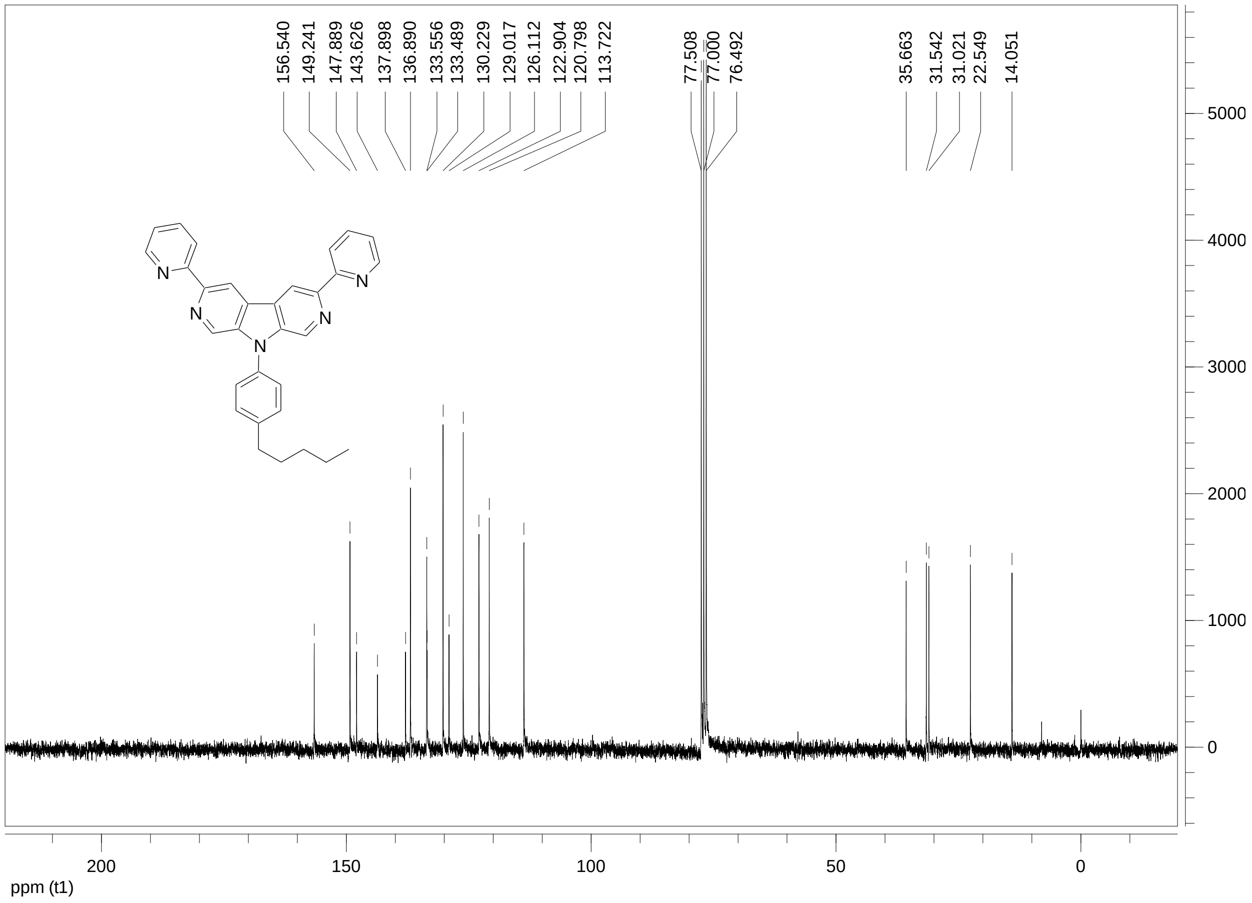
156.540
149.241
147.889
143.626
137.898
136.890
133.556
133.489
130.229
129.017
126.112
122.904
120.798
113.722
77.508
77.000
76.492
35.663
31.542
31.021
22.549
14.051
5000
4000
3000
2000
1000
0
200
150
100
50
0
ppm (t1)
N
N
N
N
N

3. Synthesis and characterization of 3-Chloro-6-(4-formylphenyl)-9-(4-pentylphenyl)-2,7-diazacarbazole (10).

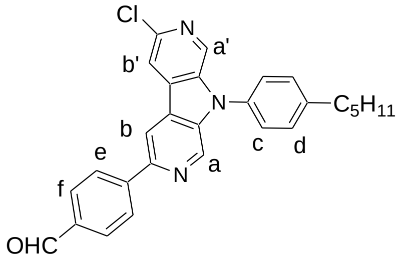

A Schlenk tube was charged with **3a** (50 mg, 0.13 mmol), **4** (58.5 mg, 0.39 mmol), $Pd_2(dba)_3$ (2.4 mg, 0.0026 mmol), SPhos (4.3 mg, 0.0104 mmol) and K_3PO_4 (55 mg, 0.26 mmol). The Schlenk tube was evacuated under vacuum, filled with argon (three times) and degassed toluene (1.5 mL) was added. The mixture was then heated at 90 °C for 24 h. After cooling, the mixture was filtered through a pad of silica gel (dichloromethane/ethyl acetate, 1:1). The filtrate was concentrated to give a residue, which was purified by column chromatography (silica gel: cyclohexane/ethyl acetate 9:1) to afford the starting material **3a** (12 mg, 24%) and **10** (25 mg, 42%) separately. Mp 141–143 °C; ^{1}H NMR (200 MHz, $CDCl_3$) δ 10.09 (s, 1H, CHO), 9.05 (s, 1H, H_a), 8.72 (s, 1H, $H_{a'}$), 8.53 (s, 1H, H_b), 8.28 (d, J = 7.5 Hz, 2H, H_e), 8.16 (s, 1H, $H_{b'}$), 8.02 (d, J = 7.5 Hz, 2H, H_f), 7.50 (m, 4H, $H_{c,d}$), 2.78 (t, J = 7 Hz, 2H, CH_2), 1.76 (m, 2H, CH_2), 1.42 (m, 4H, CH_2), 0.96 (m, 3H, CH_3); ^{13}C NMR (63 MHz, $CDCl_3$) δ 192.2, 147.5, 145.8, 145.3, 144.5, 142.0, 138.0, 137.3, 136.2, 135.0, 134.2, 133.1, 130.8, 130.6, 128.3, 127.5, 126.4, 116.1, 113.4, 36.0, 31.8, 31.3, 22.8, 14.3; MS (70 eV) m/z (%): 453 (100, M^+), 396 (70); HRMS m/z: calcd for $C_{28}H_{25}ClN_3O$ $(M + H)^+$ 454.1681, found: 454.1692.

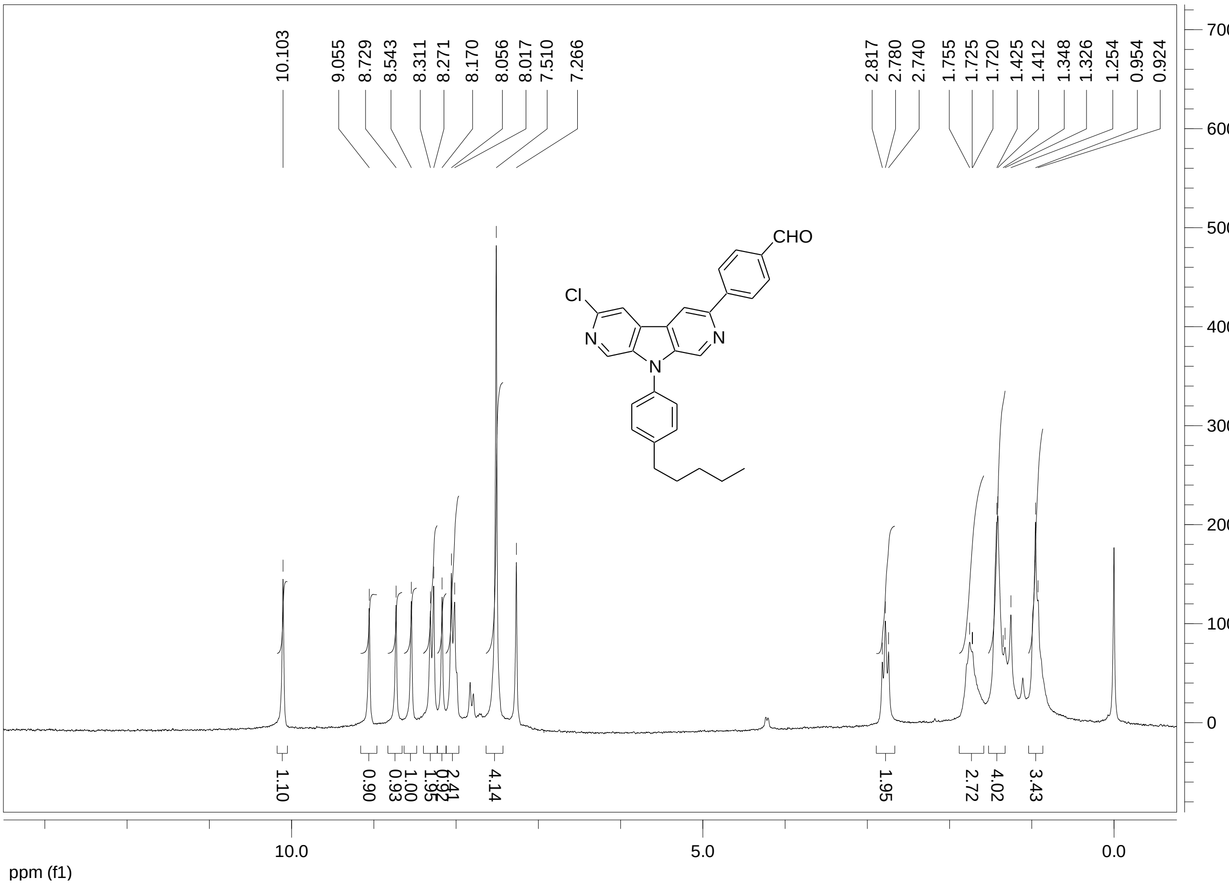
10.103
9.055
8.729
8.543
8.311
8.271
8.170
8.056
8.017
7.510
7.266
2.817
2.780
2.740
1.755
1.725
1.720
1.425
1.412
1.348
1.326
1.254
0.954
0.924
CHO
Cl
N
N
N
1.10
0.90
0.93
1.00
1.95
4.14
1.95
2.72
4.02
3.43
10.0
5.0
0.0
ppm (f1)

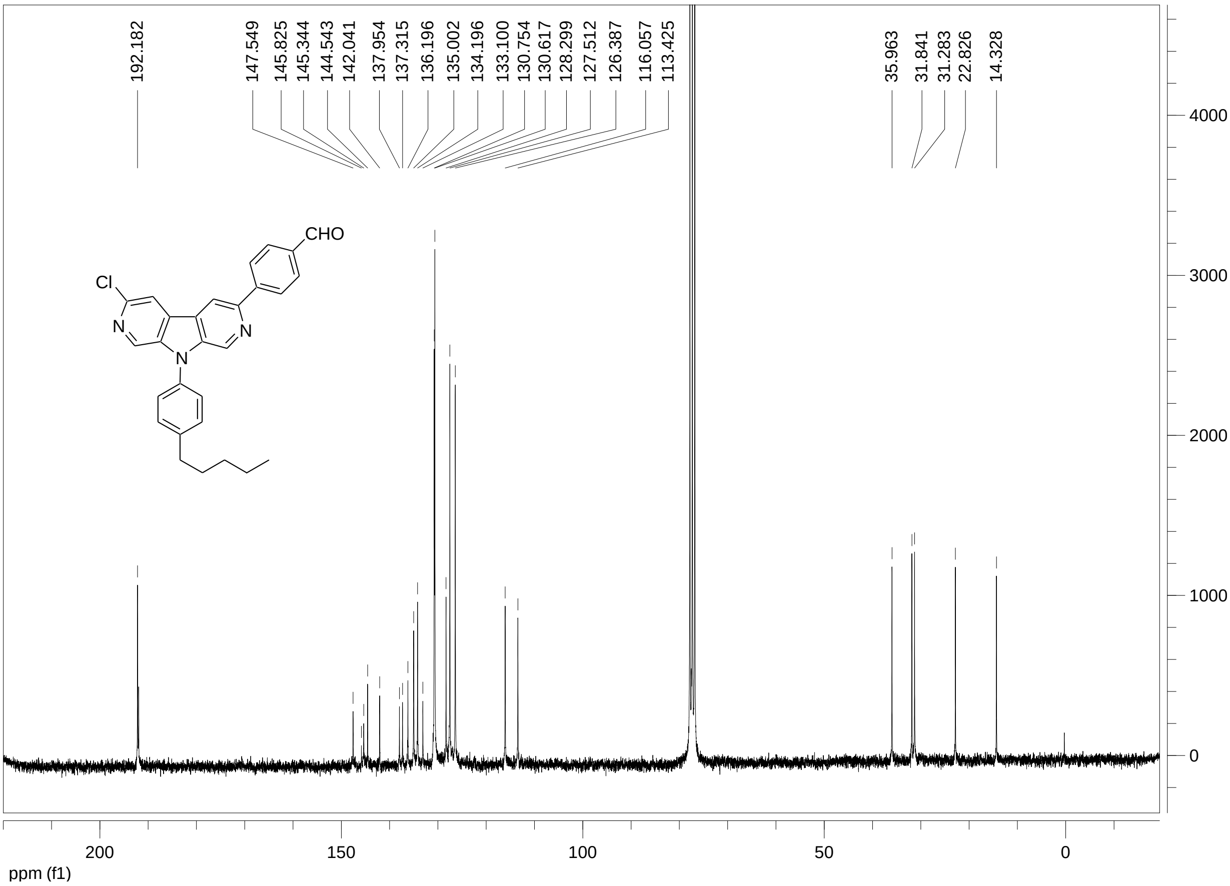
192.182
147.549
145.825
145.344
144.543
142.041
137.954
137.315
136.196
135.002
134.196
133.100
130.754
130.617
128.299
127.512
126.387
116.057
113.425
35.963
31.841
31.283
22.826
14.328
CHO
Cl
N
N
N
200
150
100
50
0
ppm (f1)

4. Synthesis and characterization of compounds 11.

To a degassed toluene solution (1.3 mL) containing $Pd(PPh_3)_4$ (10 mg, 0.0085 mmol) and **1b** (78 mg, 0.17 mmol), degassed solutions of phenylboronic acid (41 mg, 0.34 mmol) in methanol (0.7 mL) and Na_2CO_3 (71 mg, 0.34 mmol) in water (0.7 mL) were successively added. After heating for 15 h at 100 °C, the reaction mixture was cooled to room temperature, extracted with ethyl acetate and dried over $MgSO_4$. After concentration, the residue was purified by chromatography on silica gel (dichloromethane/cyclohexane 7:3) to give compound **11a** (75 mg, 95%). Compound **11b** was obtained similarly in 94% yield (89 mg) by using 4-methylsulfanylphenylboronic acid (56 mg, 0.33 mmol).

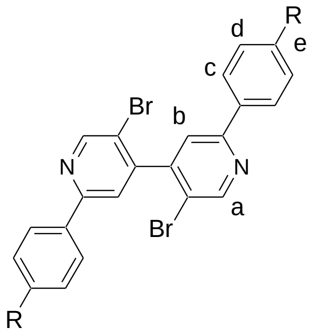

R = H **11a**
R = SMe **11b**

5,5'-Dibromo-2,2'-diphenyl-4,4'-bipyridine (11a). Mp 247–249 °C; ^{1}H NMR (250 MHz, d_6-DMSO) δ 9.00 (s, 2H, H_a), 8.18 (m, 3H, $H_{b,c}$), 7.50 (m, 3H, $H_{d,e}$); ^{13}C NMR (63 MHz, d_6-DMSO) δ 155.3, 151.2, 147.4, 136.9, 129.7, 128.8, 126.6, 121.6, 118.9; MS (70 eV) m/z (%): 466 (75, M^+), 387 (35), 77 (100), 51 (72); HRMS m/z$^+$ calcd for $C_{22}H_{15}Br_2N_2$ $(M + H)^+$ 464.9597, found: 464.9631.

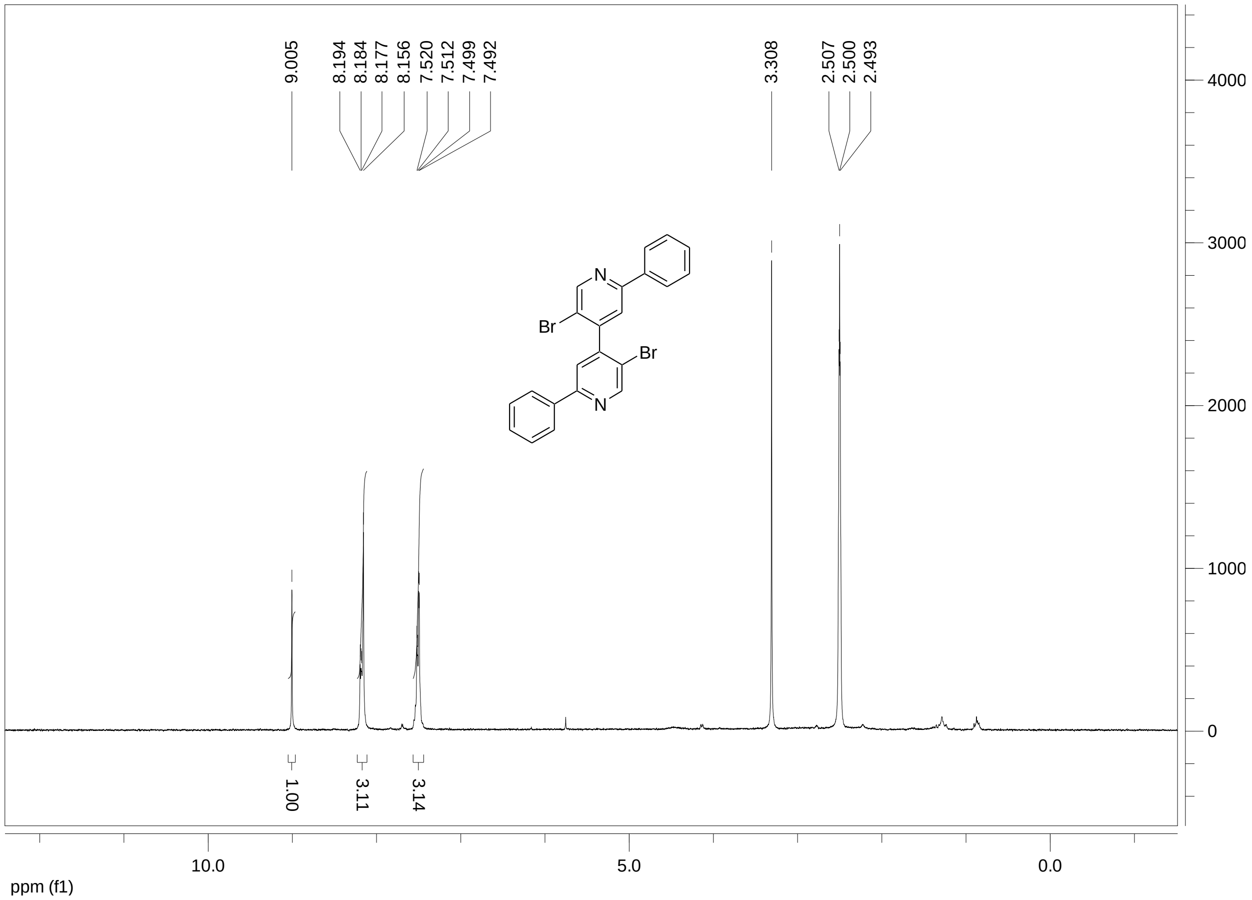
9.005
8.194
8.184
8.177
8.156
7.520
7.512
7.499
7.492
3.308
2.507
2.500
2.493
Br
N
Br
N
1.00
3.11
3.14
4000
3000
2000
1000
0
10.0
5.0
0.0
ppm (f1)

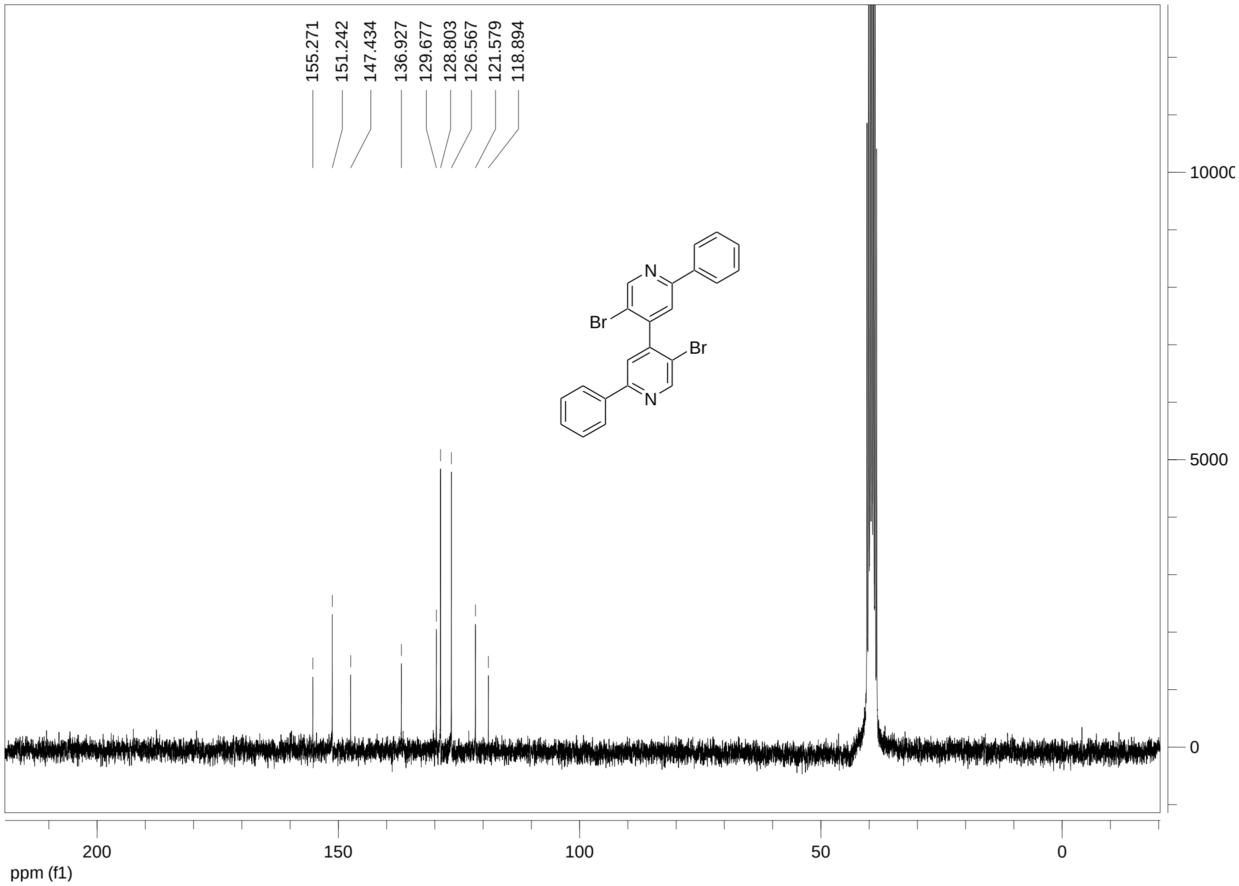
155.271
151.242
147.434
136.927
129.677
128.803
126.567
121.579
118.894
Br
N
Br
N
10000
5000
0
200
150
100
50
0
ppm (f1)

5,5’-Dibromo-2,2’-bis-(4-methylsulfanylphenyl)-4,4’-bipyridine (11b). Mp 215–216 °C; ^{1}H NMR (250 MHz, $CDCl_3$) δ 8.89 (s, 2H, H_a), 7.95 (d, *J* = 8.2 Hz, 4H, H_c), 7.61 (s, 2H, H_b), 7.33 (d, *J* = 8.2 Hz, 4H, H_d), 2.53 (s, 6H, SCH_3); ^{13}C NMR (63 MHz, $CDCl_3$) δ 155.9, 152.1, 147.5, 141.1, 127.1, 126.3, 120.9; MS (70 eV) m/z (%): 558 (100, M^+), 279 (10); HRMS m/z: calcd for $C_{24}H_{19}Br_2N_2S_2$ $(M + H)^+$ 556.9351, found: 556.9362.

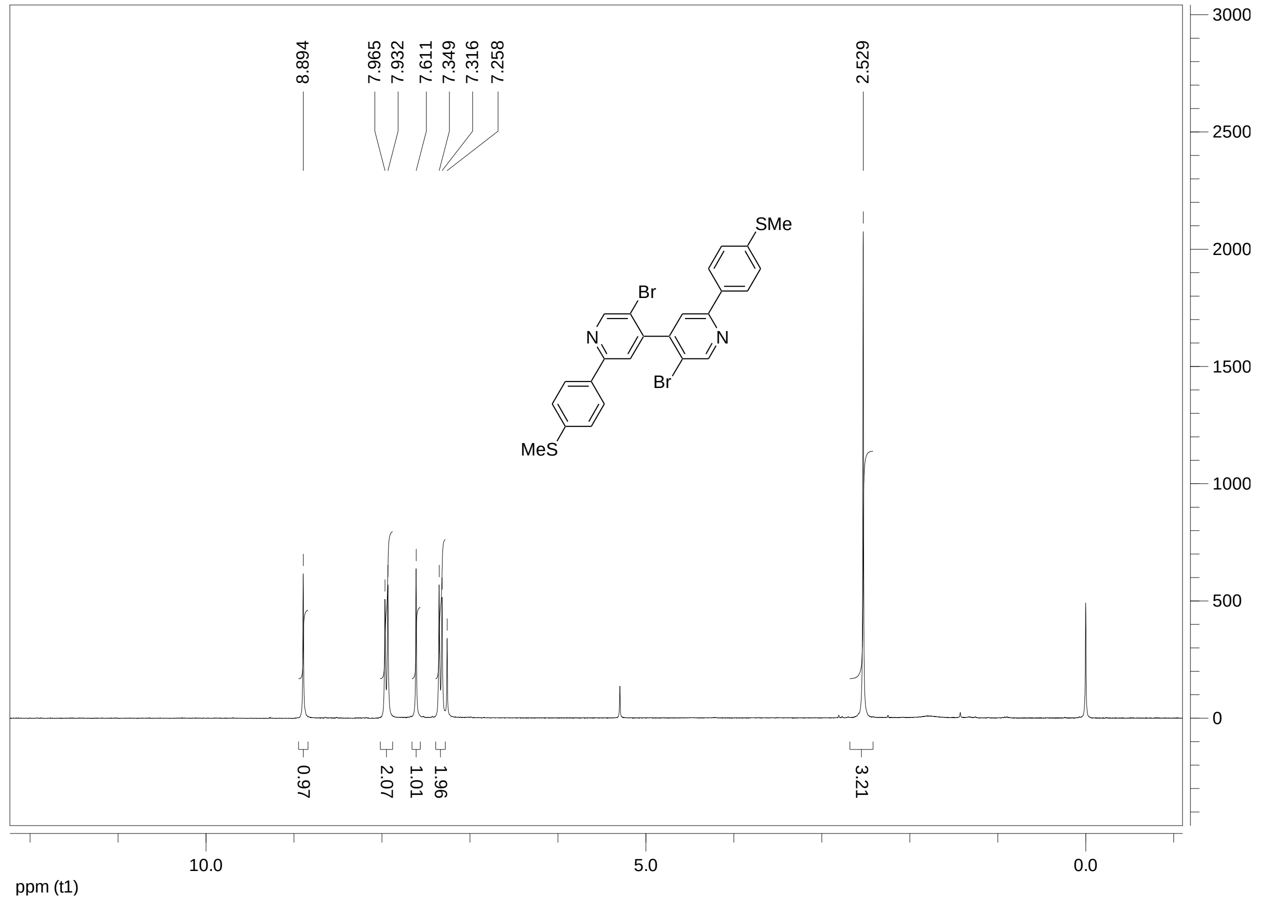

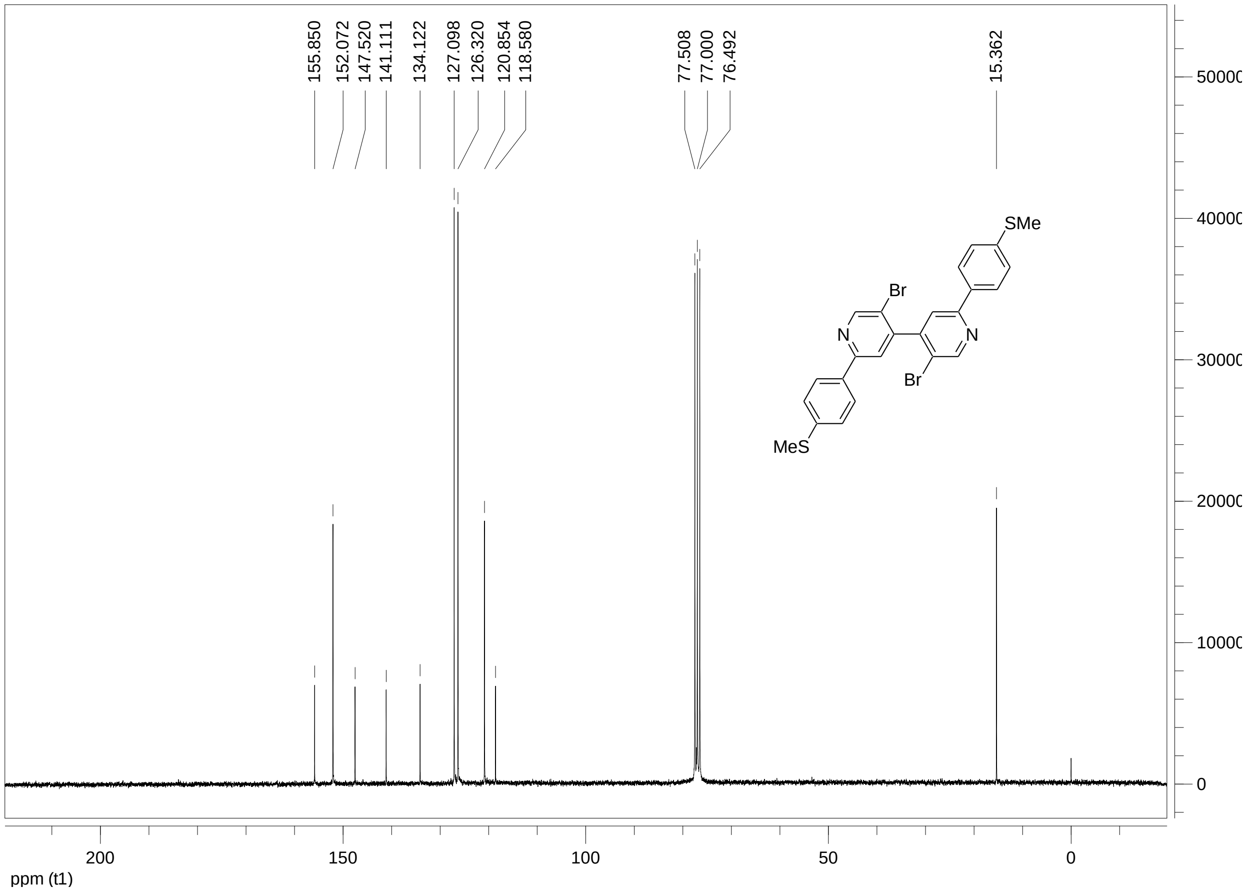
155.850
152.072
147.520
141.111
134.122
127.098
126.320
120.854
118.580
77.508
77.000
76.492
15.362
SMe
Br
N
N
Br
MeS
50000
40000
30000
20000
10000
0
200
150
100
50
0
ppm (t1)

5. Synthesis and characterization of compounds 12.

These were obtained according to the general procedure of the double N-arylation reaction.

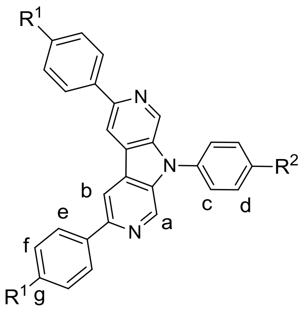

R^1 = H, R^2 = C_5H_{11} **12a**
R^1 = SMe, R^2 = C_5H_{11} **12b**
R^1 = SMe, R^2 = OMe **12c**

3,6-Diphenyl-9-(4-pentylphenyl)-2,7-diazacarbazole (12a). 11a (35 mg, 0.075 mmol), **6a** (12.2 mg, 0.075 mmol), $Pd_2(dba)_3$ (6.9 mg, 0.0075 mmol), XPhos (10.7 mg, 0.0225 mmol), NaO*t*-Bu (21.6 mg, 0.225 mmol). **12a**: m = 27 mg, 77% yield. Mp 131–133 °C; ^{1}H NMR (250 MHz, $CDCl_3$) δ 9.04 (s, 2H, H_a), 8.52 (s, 2H, H_b), 8.12 (d, *J* = 7.5 Hz, 4H, H_e), 7.56 (d, *J* = 8.3 Hz, 2H, H_c), 7.51 (d, *J* = 8.3 Hz, 2H, H_d), 7.49 (t, *J* = 7.5 Hz, 4H, H_f), 7.40 (t, *J* = 7.5 Hz, 2H, H_g), 2.77 (t, *J* = 7.5 Hz, 2H, CH_2), 1.76 (quint, *J* = 7.5 Hz, 2H, CH_2), 1.40 (m, 4H, CH_2), 0.96 (t, *J* = 7.5 Hz, 2H, CH_3); ^{13}C NMR (63 MHz, $CDCl_3$) δ 148.9, 143.5, 139.9, 136.9, 134.2, 133.5, 130.2, 128.8, 128.6, 128.1, 126.8, 126.0, 112.1, 35.7, 31.5, 31.0, 22.6, 14.1; MS (70 eV) m/z (%): 467 (100, M^+), 410 (60); HRMS m/z: calcd for $C_{33}H_{30}N_3$ $(M + H)^+$ 468.2434, found: 468.2474.

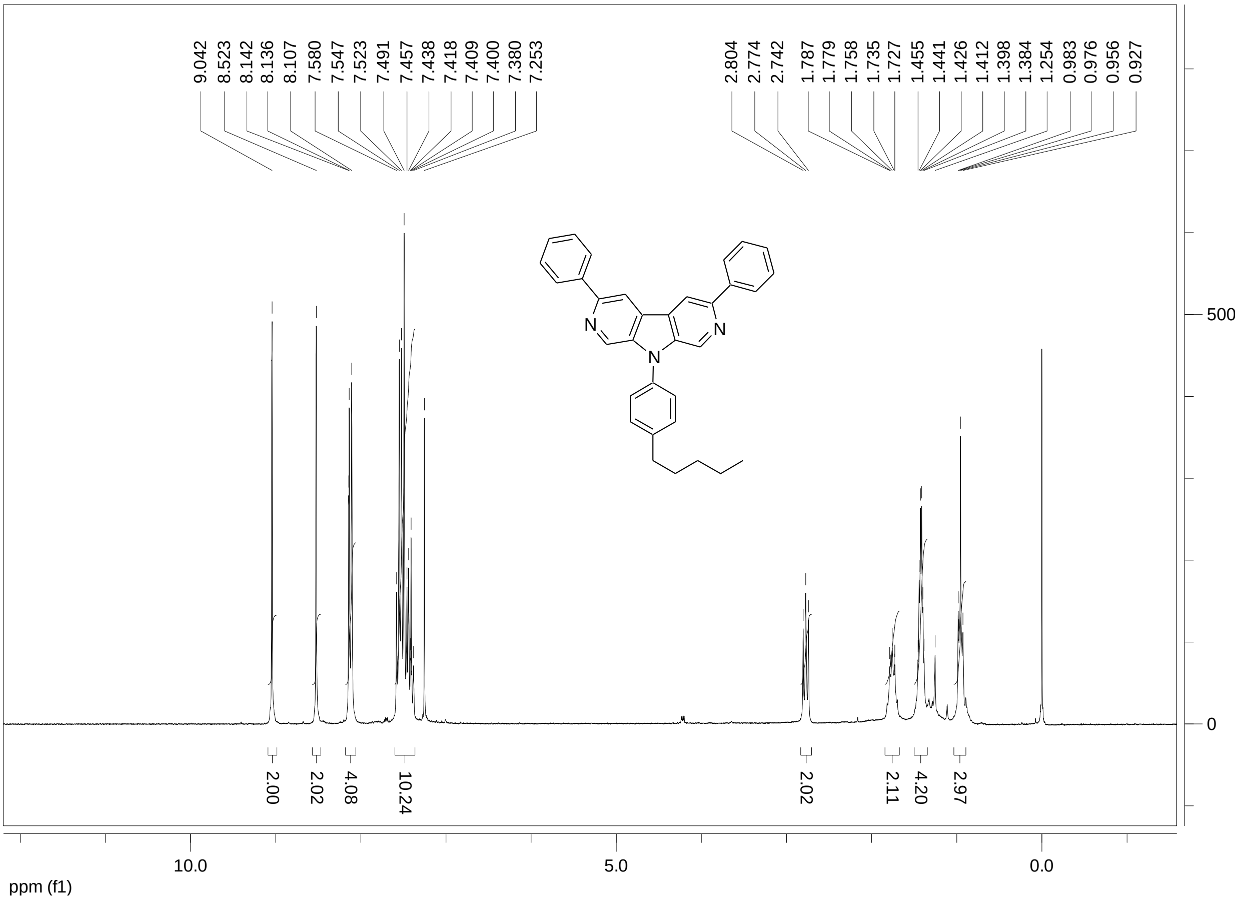
9.042
8.523
8.142
8.136
8.107
7.580
7.547
7.523
7.491
7.457
7.438
7.418
7.409
7.400
7.380
7.253
2.804
2.774
2.742
1.787
1.779
1.758
1.735
1.727
1.455
1.441
1.426
1.412
1.398
1.384
1.254
0.983
0.976
0.956
0.927
N
N
N
500
0
2.00
2.02
4.08
10.24
2.02
2.11
4.20
2.97
10.0
5.0
0.0
ppm (f1)

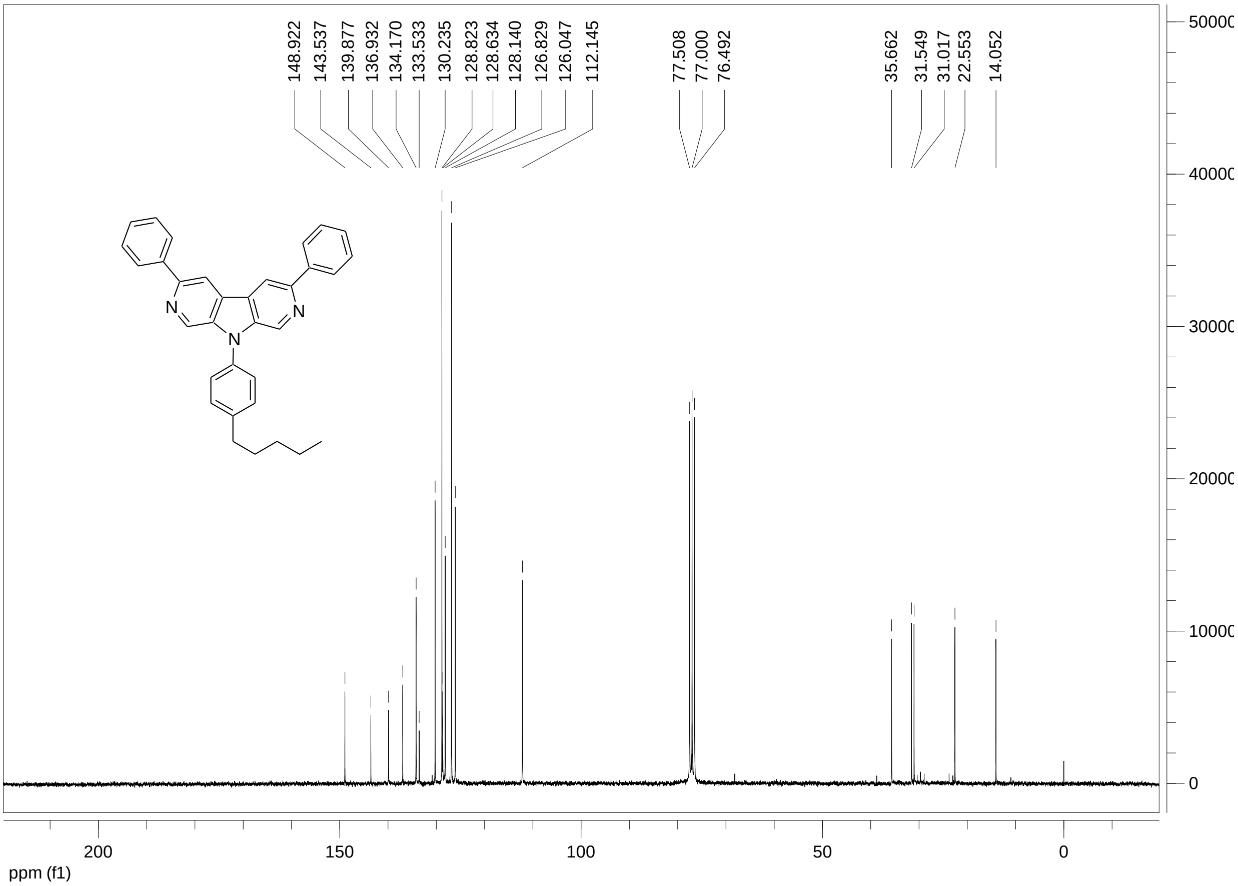
148.922
143.537
139.877
136.932
134.170
133.533
130.235
128.823
128.634
128.140
126.829
126.047
112.145
77.508
77.000
76.492
35.662
31.549
31.017
22.553
14.052
N
N
N
200
150
100
50
0
ppm (f1)

3,6-Bis-(4-methylsulfanylphenyl)-9-(4-pentylphenyl)-2,7-diazacarbazole (12b). **11b** (42 mg, 0.075 mmol), **6a** (12.2 mg, 0.075 mmol), $Pd_2(dba)_3$ (6.9 mg, 0.0075 mmol), XPhos (10.7 mg, 0.0225 mmol), NaO*t*-Bu (21.6 mg, 0.225 mmol). **12b**: m = 34 mg, 81% yield; Mp 166–168 °C; ^{1}H NMR (250 MHz, $CDCl_3$) δ 9.00 (s, 2H, H_a), 8.45 (s, 2H, H_b), 8.04 (d, *J* = 8.3 Hz, 4H, H_e), 7.53 (d, *J* = 8.3 Hz, 2H, H_c), 7.46 (d, *J* = 8.3 Hz, 2H, H_d), 7.38 (d, *J* = 8.3 Hz, 4H, H_f), 2.76 (t, *J* = 7.5 Hz, 2H, CH_2), 2.55 (s, 6H, SCH_3), 1.75 (quint, *J* = 7.5 Hz, 2H, CH_2), 1.41 (m, 4H, CH_2), 0.95 (t, *J* = 7.5 Hz, 2H, CH_3); ^{13}C NMR (63 MHz, $CDCl_3$) δ 148.3, 143.5, 138.6, 136.8, 136.7, 134.1, 133.5, 130.2, 128.6, 127.1, 126.8, 126.0, 111.6, 35.6, 31.5, 31.0, 22.5, 15.8, 14.0; MS (70 eV) m/z (%): 559 (100, M^+), 280 (15); HRMS m/z: calcd for $C_{35}H_{34}N_3S_2$ $(M + H)^+$ 560.2189, found: 560.2196.

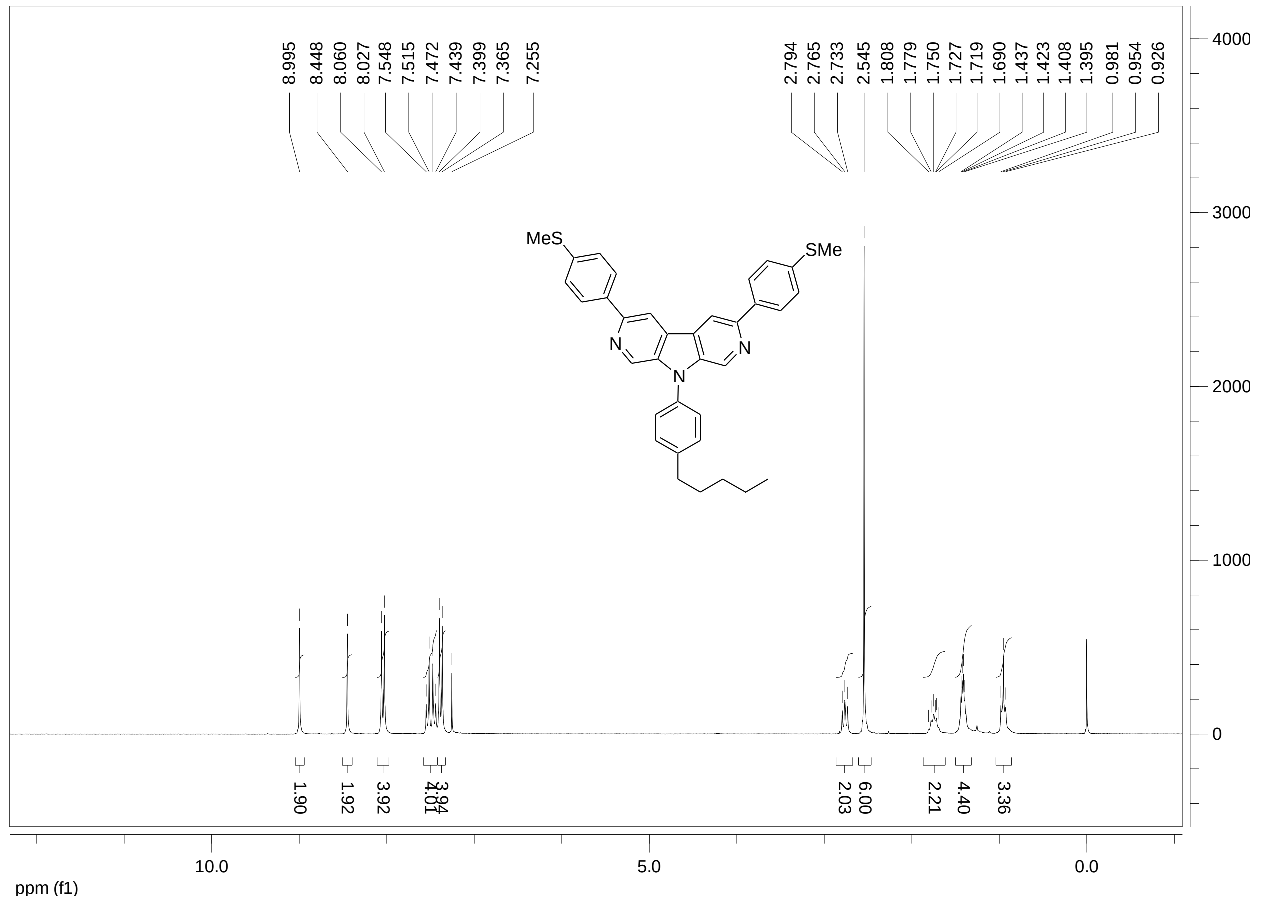

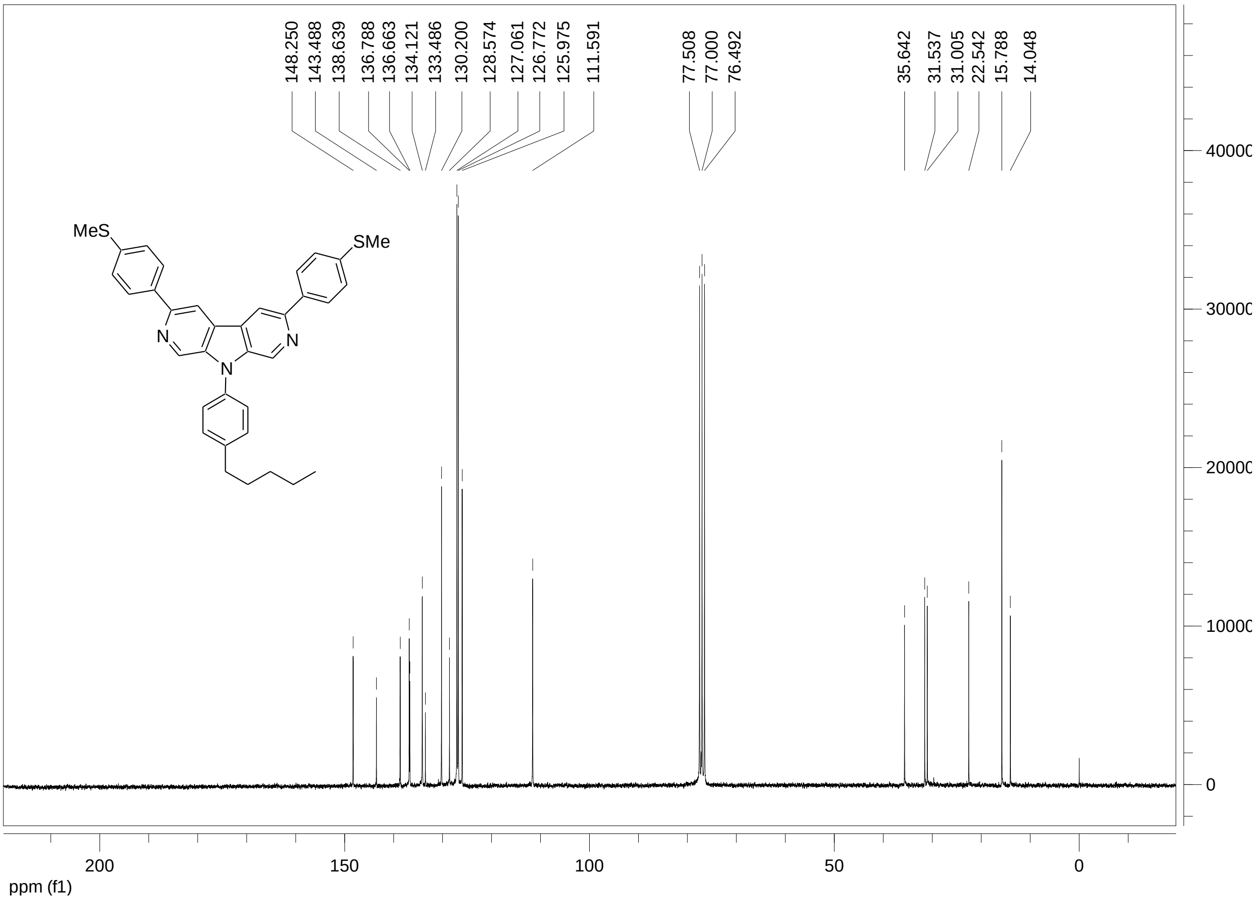
148.250
143.488
138.639
136.788
136.663
134.121
133.486
130.200
128.574
127.061
126.772
125.975
111.591
77.508
77.000
76.492
35.642
31.537
31.005
22.542
15.788
14.048
MeS
SMe
N
N
N
40000
30000
20000
10000
0
200
150
100
50
0
ppm (f1)

3,6-Bis-(4-methylsulfanylphenyl)9-(4-methoxyphenyl)-2,7-diazacarbazole (12c). 11b (64 mg, 0.1 mmol), **6b** (12.7 mg, 0.1 mmol), $Pd_2(dba)_3$ (9.2 mg, 0.01 mmol), XPhos (14.3 mg, 0.03 mmol), NaO*t*-Bu (29 mg, 0.3 mmol). **12c**: m = 38 mg, 73% yield; Mp > 250 °C; ^{1}H NMR (200 MHz, $CDCl_3$) δ 8.94 (d, *J* = 0.8 Hz, 2H, H_a), 8.47 (d, *J* = 0.8 Hz, 2H, H_b), 8.05 (d, *J* = 8.5 Hz, 4H, H_e), 7.54 (d, *J* = 9 Hz, 2H, H_c), 7.39 (d, *J* = 8.5 Hz, 4H, H_f), 7.17 (d, *J* = 9 Hz, 2H, H_d), 3.95 (s, 3H, OCH_3), 2.55 (s, 6H, SCH_3); ^{13}C NMR (63 MHz, $CDCl_3$) δ 159.5, 148.3, 138.6, 137.1, 136.7, 134.0, 128.5, 128.4, 127.7, 127.7, 126.8, 115.5, 111.6, 55.7, 15.8; HRMS m/z: calcd for $C_{31}H_{26}N_3OS_2$ $(M + H)^+$ 520.1512, found: 520.1492.

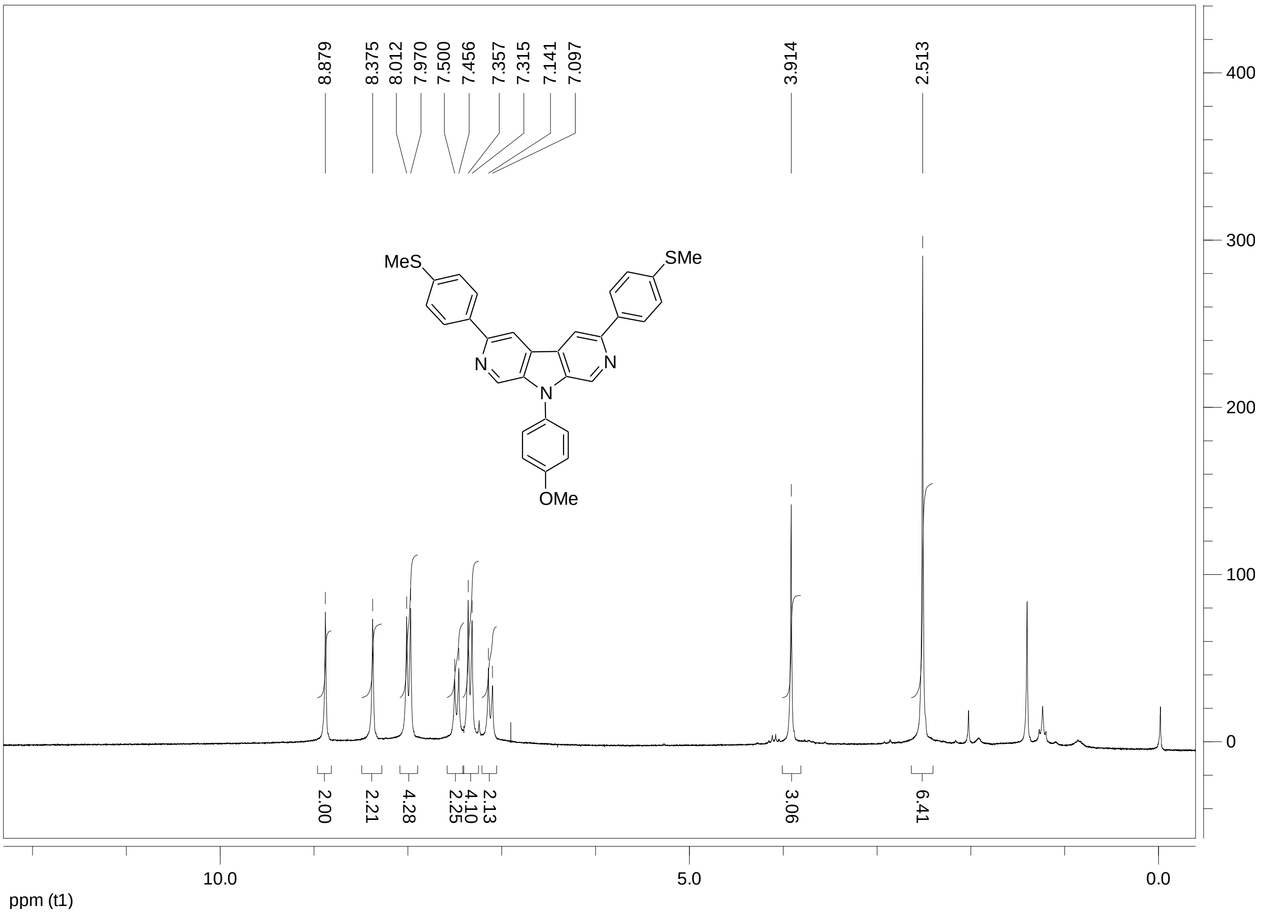

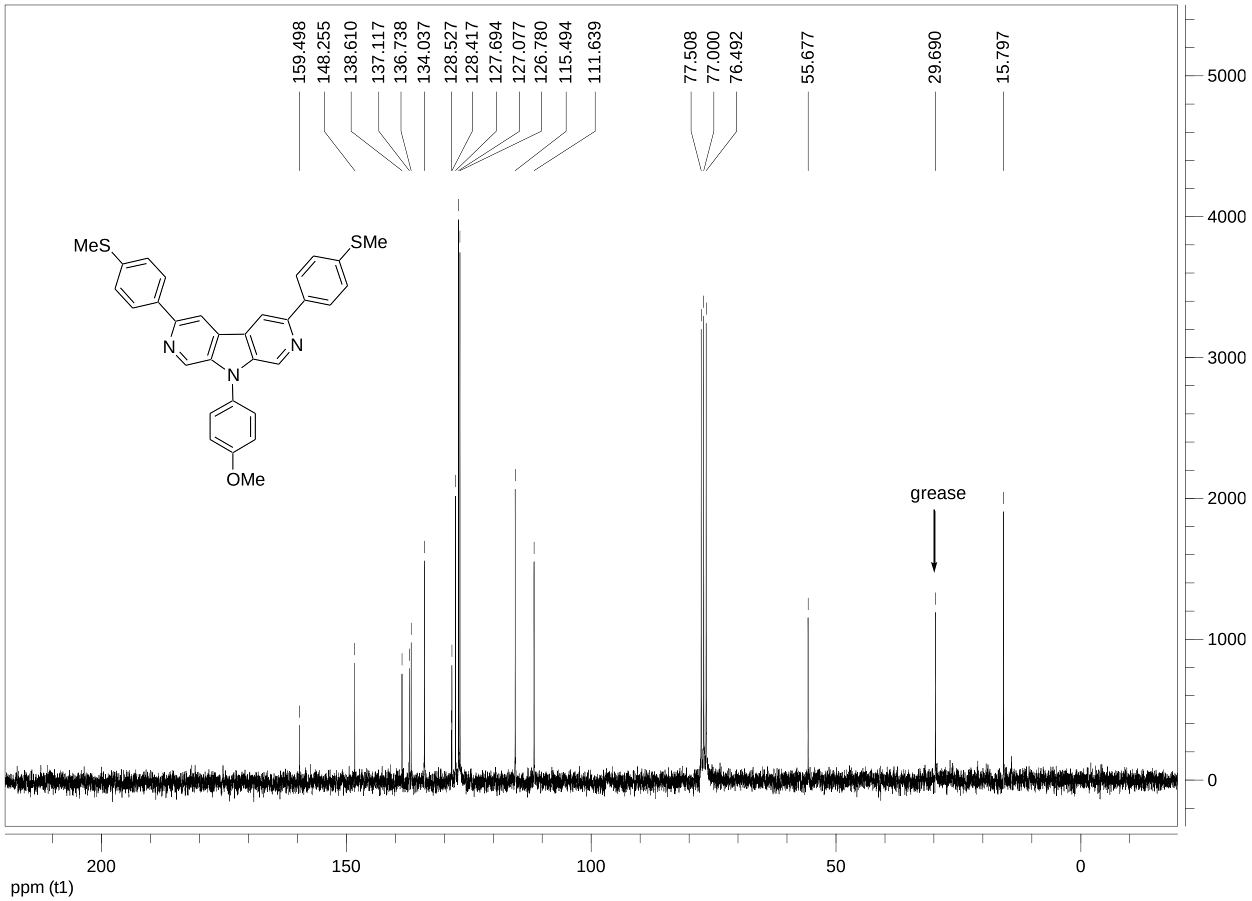
159.498
148.255
138.610
137.117
136.738
134.037
128.527
128.417
127.694
127.077
126.780
115.494
111.639
77.508
77.000
76.492
55.677
29.690
15.797
MeS
SMe
N
N
N
OMe
grease
5000
4000
3000
2000
1000
0
200
150
100
50
0
ppm (t1)

6. X-ray structure determination

General. The crystal structure analysis was performed at low temperature (T = 110 K) on an Oxford Diffraction SuperNova CCD diffractometer by using Cu Kα radiation (λ = 1.5418 Å). The structures were solved by direct methods with the program SIR92 (Altomare, A.; Cascarano, G.; Giacovazzo, C.; Guagliardi, A. *J. Appl. Crystallogr.* **1993**, *26*, 343) and full-matrix least-square refinements on F^2 in SHELXL-97 (Sheldrick, G. M. SHELXL-97, University of Göttingen, Germany, **1997**) were performed with anisotropic displacements for non-H atoms. Hydrogen atoms were located in difference Fourier maps and refined isotropically according to a riding model.

Crystallographic data of compound 3b

Residual density was found within the channels parallel to [010], but refinement of the disordered *n*-hexane molecules was not successful; this is due to the fact that the *n*-hexane molecules are ~2.7 times longer than the crystallographic *b* axis. The final model was then constructed by inserting carbon atoms with partial occupancies into these channels. CCDC 818528 contains the detailed crystallographic data. These data can be obtained free of charge from the Cambridge Crystallographic Data Centre at www.ccdc.cam.ac.ik/data_request/cif.

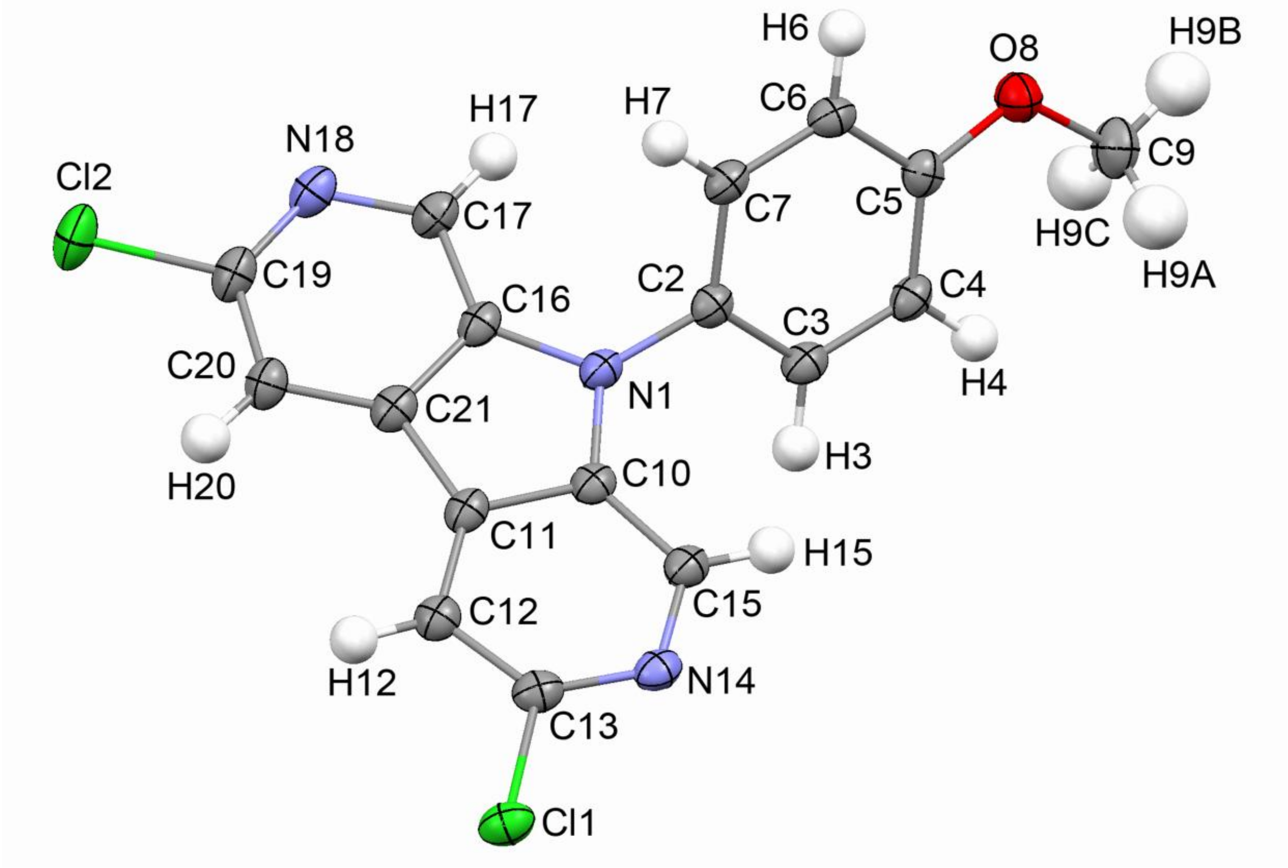

ORTEP-3 view of **3b** in space group *C*2/*c*. Atomic displacement ellipsoids are drawn at the 50% probability level. Disordered carbon atoms of the *n*-hexane solvent molecule are not shown.

Table S1. Crystal data and structure refinement for **3b**.

Identification code	**3b**	
Empirical formula	C18.5 H11 Cl2 N3 O	
Formula weight	362.21	
Temperature	110(2) K	
Wavelength	1.54184 Å	
Crystal system	Monoclinic	
Space group	*C* 2/*c*	
Unit cell dimensions	*a* = 30.9380(7) Å	α= 90°.
	b = 3.88160(10) Å	β= 108.837(2)°.
	c = 28.1690(6) Å	γ = 90°.
Volume	3201.61(13) $Å^3$	
Z	8	
Density (calculated)	1.503 Mg/m^3	
Absorption coefficient	3.710 mm^{-1}	
F(000)	1480	
Crystal size	0.1267 x 0.0689 x 0.0272 mm^3	
Theta range for data collection	3.32 to 76.30°.	
Index ranges	-38<=h<=36, -4<=k<=4, -34<=l<=34	
Reflections collected	10777	
Independent reflections	3340 [R(int) = 0.0167]	
Completeness to theta = 76.30°	99.9 %	
Absorption correction	Analytical	
Max. and min. transmission	0.907 and 0.725	
Refinement method	Full-matrix least-squares on F^2	
Data / restraints / parameters	3340 / 24 / 237	
Goodness-of-fit on F^2	1.101	
Final R indices [I>2sigma(I)]	R1 = 0.0410, wR2 = 0.1310	
R indices (all data)	R1 = 0.0454, wR2 = 0.1354	
Largest diff. peak and hole	0.893 and -0.237 e.$Å^{-3}$	

Crystallographic data of compound 12c

Residual density was found within the channels parallel to [100], but refinement of the disordered chloroform molecules was not successful. The final model was then constructed by inserting chlorine atoms with partial occupancies into these channels. CCDC 818527 contains the detailed crystallographic data. These data can be obtained free of charge from the Cambridge Crystallographic Data Centre at www.ccdc.cam.ac.ik/data_request/cif.

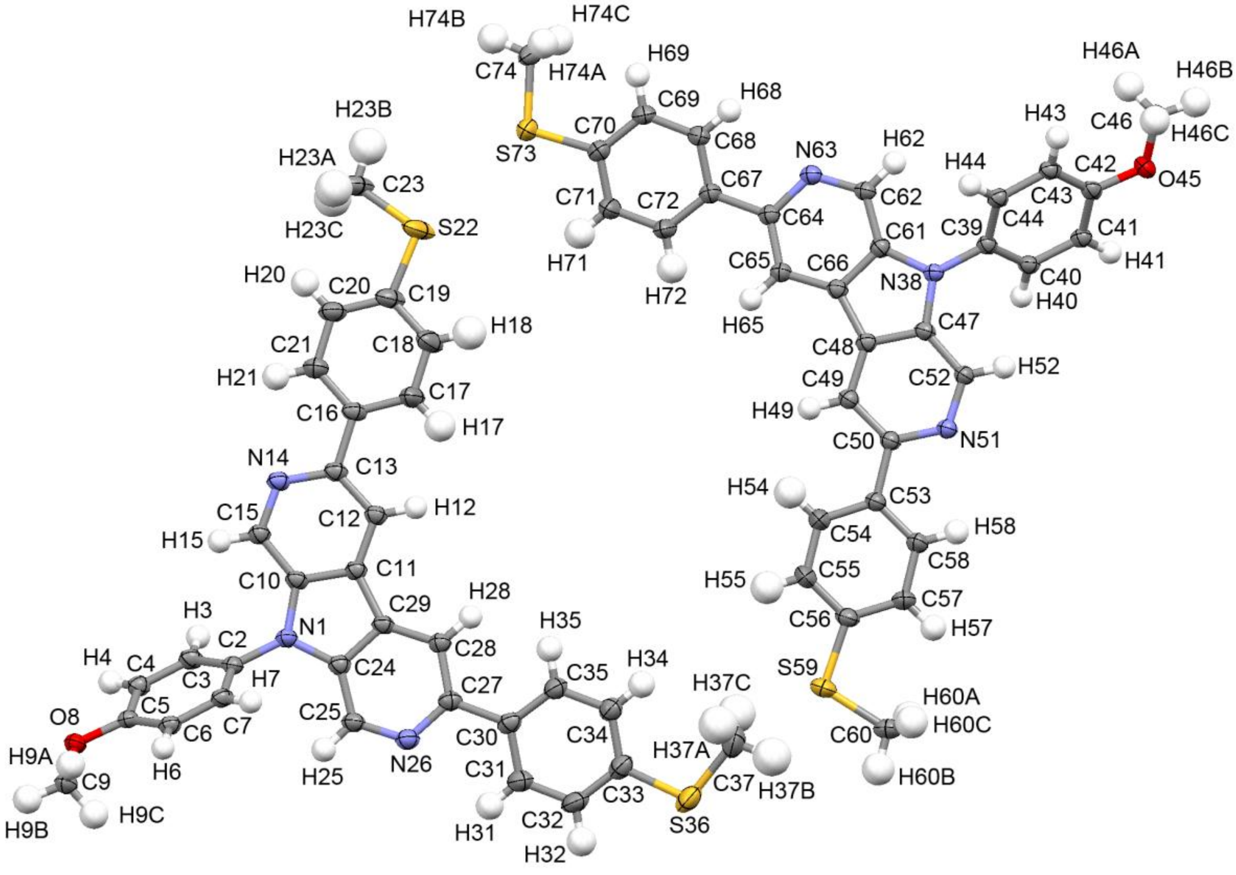

ORTEP-3 view of **12c** in space group *P*-1. Atomic displacement ellipsoids are drawn at the 50% probability level. Disordered chlorine atoms of the chloroform molecules are not show.

Table S2. Crystal data and structure refinement for **12c.**

Identification code	**12c**	
Empirical formula	C31 H25 Cl0.85 N3 O S2	
Formula weight	549.92	
Temperature	110(2) K	
Wavelength	1.54184 Å	
Crystal system	Triclinic	
Space group	*P* -1	
Unit cell dimensions	a = 9.64985(16) Å	α= 99.0852(12)°.
	b = 16.5066(2) Å	β= 91.9074(13)°.
	c = 17.1719(3) Å	γ = 93.8073(12)°.
Volume	2692.30(7) Å^3	
Z	4	
Density (calculated)	1.357 Mg/m^3	
Absorption coefficient	2.817 mm^{-1}	
F(000)	1145.8	
Crystal size	0.1661 x 0.1078 x 0.0735 mm^3	
Theta range for data collection	3.45 to 76.43°.	
Index ranges	-12<=h<=12, -20<=k<=20, 0<=l<=21	
Reflections collected	44453	
Independent reflections	11242 [R(int) = 0.0250]	
Completeness to theta = 76.43°	99.5 %	
Absorption correction	Analytical	
Max. and min. transmission	0.862 and 0.725	
Refinement method	Full-matrix least-squares on F^2	
Data / restraints / parameters	11242 / 0 / 717	
Goodness-of-fit on F^2	1.067	
Final R indices [I>2sigma(I)]	R1 = 0.0602, wR2 = 0.1638	
R indices (all data)	R1 = 0.0671, wR2 = 0.1689	
Largest diff. peak and hole	1.020 and -0.825 e.Å^{-3}	
